# Dolutegravir and Folic Acid Interaction during Neural System Development in Zebrafish Embryos

**DOI:** 10.3390/ijms25094640

**Published:** 2024-04-24

**Authors:** Daniela Zizioli, Eugenia Quiros-Roldan, Sara Ferretti, Luca Mignani, Giorgio Tiecco, Eugenio Monti, Francesco Castelli, Isabella Zanella

**Affiliations:** 1 Department of Molecular and Translational Medicine, University of Brescia, 25123 Brescia, Italy; daniela.zizioli@unibs.it (D.Z.); s.ferretti002@studenti.unibs.it (S.F.); luca.mignani1@unibs.it (L.M.); eugenio.monti@unibs.it (E.M.); isabella.zanella@unibs.it (I.Z.); 2 Unit of Infectious and Tropical Diseases, Department of Clinical and Experimental Sciences, University of Brescia and ASST Spedali Civili di Brescia, 25123 Brescia, Italy; g.tiecco@unibs.it (G.T.); francesco.castelli@unibs.it (F.C.); 3 Cytogenetics and Molecular Genetics Laboratory, Diagnostic Department, ASST Spedali Civili di Brescia, 25123 Brescia, Italy

**Keywords:** dolutegravir, integrase strand transfer inhibitors, neurotoxicity, developmental safety, fetal anomalies, neural tube defects, behavior, zebrafish embryo, dopaminergic neurons

## Abstract

Dolutegravir (DTG) is one of the most prescribed antiretroviral drugs for treating people with HIV infection, including women of child-bearing potential or pregnant. Nonetheless, neuropsychiatric symptoms are frequently reported. Early reports suggested that, probably in relation to folic acid (FA) shortage, DTG may induce neural tube defects in infants born to women taking the drug during pregnancy. Subsequent reports did not definitively confirm these findings. Recent studies in animal models have highlighted the association between DTG exposure in utero and congenital anomalies, and an increased risk of neurologic abnormalities in children exposed during in utero life has been reported. Underlying mechanisms for DTG-related neurologic symptoms and congenital anomalies are not fully understood. We aimed to deepen our knowledge on the neurodevelopmental effects of DTG exposure and further explore the protective role of FA by the use of zebrafish embryos. We treated embryos at 4 and up to 144 h post fertilization (hpf) with a subtherapeutic DTG concentration (1 μM) and observed the disruption of the anterior–posterior axis and several morphological malformations in the developing brain that were both prevented by pre-exposure (2 hpf) and rescued by post-exposure (10 hpf) with FA. By whole-mount in situ hybridization with riboprobes for genes that are crucial during the early phases of neurodevelopment (*ntl*, *pax2a*, *ngn1*, *neurod1*) and by in vivo visualization of the transgenic Tg(*ngn1*:EGFP) zebrafish line, we found that DTG induced severe neurodevelopmental defects over time in most regions of the nervous system (notochord, midbrain–hindbrain boundary, eye, forebrain, midbrain, hindbrain, spinal cord) that were mostly but not completely rescued by FA supplementation. Of note, we observed the disruption of *ngn1* expression in the dopaminergic regions of the developing forebrain, spinal cord neurons and spinal motor neuron projections, with the depletion of the tyrosine hydroxylase (TH)^+^ dopaminergic neurons of the dorsal diencephalon and the strong reduction in larvae locomotion. Our study further supports previous evidence that DTG can interfere with FA pathways in the developing brain but also provides new insights regarding the mechanisms involved in the increased risk of DTG-associated fetal neurodevelopmental defects and adverse neurologic outcomes in in utero exposed children, suggesting the impairment of dopaminergic pathways.

## 1. Introduction

Dolutegravir (DTG), an HIV integrase strand transfer inhibitor (INSTI), is one of the most commonly prescribed antiretroviral drugs around the world for treating people with HIV infection (PWH), both naïve and treatment-experienced patients. DTG works by inhibiting the HIV integrase after binding to the active site of the enzyme, blocking the strand transfer step and further integration of HIV retroviral DNA into the host cell, thereby inhibiting viral activity [1].

At present, DTG-containing regimens account for 40–50% of the antiretroviral therapy of patients with HIV infection [2], since DTG is considered one of the safest antiretroviral drugs against HIV. However, reports from clinical practice about the high rate of neuropsychiatric adverse events (including sleep disorders, depression, anxiety, and suicidal behaviors) in patients treated with DTG that can lead to treatment discontinuation have raised questions on the tolerability of DTG-based regimens [1,3]. The underlying mechanisms for DTG-related neuropsychiatric symptoms are however not fully understood.

Early reports also suggested that DTG has potential teratogenic effects during pregnancy, such as the induction of neural tube defects (NTDs) [4], although subsequent reports have not further confirmed these findings [5,6,7]. Notwithstanding, a higher risk of pregnancy loss was described among women exposed to DTG, compared to other antiretroviral drugs [7]. Moreover, data from HIV-uninfected children, but HIV/DTG-exposed during their in utero life, suggested an increased risk of neurological DTG-related damage [8]. Currently, the DTG drug label still contains information warning health-care providers about potential fetal toxicity, and despite those findings, DTG is prescribed for pregnant women with HIV infection when the benefits outweigh the risks [9].

Some hypotheses have been suggested to explain the potential DTG teratogenicity. First, DTG interferes with the folic acid (FA) pathways. By using in vitro and in vivo models, Cabrera and colleagues [10] were the first ones to describe that DTG, by acting as a partial antagonist of folate receptor 1 (FOLR1), could impair the transplacental transport of FA, and they showed that DTG teratogenic damage could be rescued by supplemental FA. Furthermore, DTG can reduce the expression of the reduced folate carrier (RFC) and the proton-coupled folate transporter (PCFT), both at the mRNA and protein levels. In pregnant mice, DTG administration was associated with an increase in both placental RFC and PCFT mRNA expression, and folate-deficient dietary conditions are accompanied by a decrease in placental folate receptor α (FRα) mRNA [11]. Notwithstanding, a recent investigation described no differences on total fetal folate levels, according to DTG exposure in mice [12]. Secondly, DTG can inhibit the activity of the matrix metalloproteinases (MMPs), a class of Zn^++^-dependent enzymes involved in the developmental processes of the central nervous system (CNS) [13], and this mechanism was also suggested as involved in the DTG-induced fetal neurodevelopment defects [14]. Whether there is an association among taking DTG during conception, folate status and congenital anomalies is a current research area of increasing interest.

Zebrafish as a vertebrate organism has proven to be an excellent model to study drug toxicity in the embryo. In spite being a non-mammalian species, many behaviors of zebrafish have been well characterized and can be analyzed automatically or manually and quantitatively or qualitatively. The zebrafish behaviors have been exploited in diverse research areas such as ethology, toxicology, pharmacology, neuroscience and genetics, being particularly useful in the study of neuroactive drugs [15]. Moreover, brain development, morphology and structure in the zebrafish share many similarities with those of mammals—such as the presence of similar structures that share the same functions and of conserved cellular populations, neurotransmitters and networks. A further advantage in using zebrafish as the animal model is that the fundamental stages of neurodevelopment in embryos are completed few days after fertilization. In contrast with mammals, the development of fish larvae, which are transparent, occurs externally, making the zebrafish CNS easily visible and accessible to study its development in real-time [16].

The aim of our study was to deepen our knowledge on the effects of DTG exposure on the crucial phases of neurodevelopment and further explore the protective role of FA by the use of zebrafish embryos as in vivo animal models, to offer a complementary approach to this topic already explored in mice.

## 2. Results

**Figure 1:**
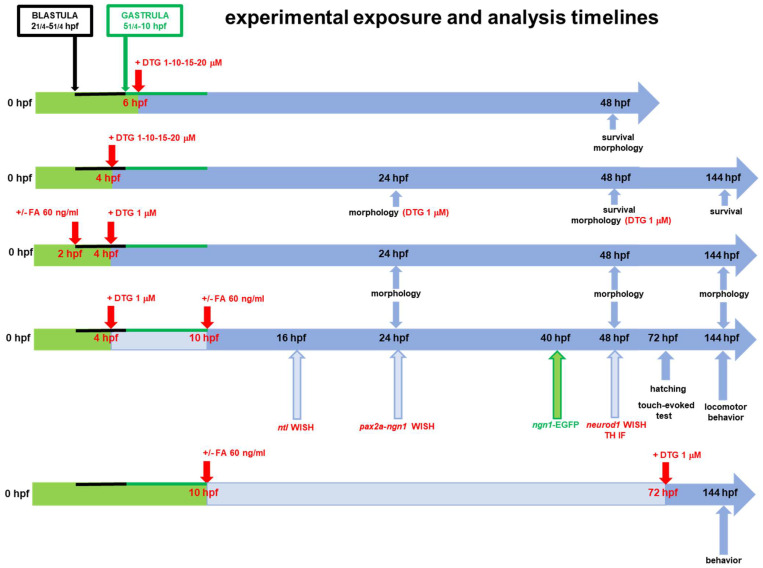
Experimental exposure to drugs and analysis timelines. Diagram depicting the timelines of the experimental procedures with performed and described analyses. Freshly spawned zebrafish eggs were collected, washed and maintained at 28 °C in Petri dishes containing fresh fish water until 2 h post fertilization (hpf) (nearby the start of blastula stage), 4 hpf (during the blastula stage), 6 hpf (nearby the onset of gastrulation) or 10 hpf (the end of gastrulation). Embryos were exposed to the drugs (dolutegravir, DTG; folic acid, FA) at the indicated times and concentrations for each experiment. Each performed experimental analysis [survival rate; gross developmental morphology; whole-mount in situ hybridization (WISH) with *no tail*, *ntl*; *paired box 2a*, *pax2a*; *neurogenin1*, *ngn1*; *neuronal differentiation 1*, *neurod1* probes; analysis of *ngn1* expression with Tg(*ngn1*:EGFP) line; hatching rate; behavior assessment by the light–dark locomotion test; touch-evoked response test; tyrosine hydroxylase immunofluorescence (TH IF)] is indicated in each timeline.

The timelines of experimental exposure and analyses performed in this work are summarized in Figure 1.

### 2.1. Early Embryonic Exposure to Dolutegravir Induces Morphological Malformations Particularly Evident in the Developing Brain Region

To explore DTG developmental toxicity, we first exposed embryos to DTG (1–10–15–20 µM) at the early gastrulation stage (6 hpf) and evaluated survival and morphology at 48 hpf (Figure 1). In these experimental conditions, low mortality was observed at any tested DTG concentration, although it was significantly higher in comparison with that in control embryos (mortality rates of 6.25%, 6.75%, 14.75% and 18%, respectively, at 1, 5, 10 and 20 µM concentrations compared to 2% in the control group) (Supplementary Figure S1A). Alive embryos presented no evident morphological malformations, confirming previously published observations [10], except for a mild pericardial edema with hemorrhage at the highest 20 µM supra-therapeutic concentration (Supplementary Figure S1B).

Then, we performed the same experiment but exposed embryos to DTG at 4 hpf, that is before gastrulation starting time (Figure 1). Similar to the previous experiments with DTG exposure at 6 hpf, for concentrations of DTG lower or equal to the human C_max_ (1 and 10 µM), mean survival rates of treated embryos were only slightly but significantly lower at 48 hpf when compared with those observed for untreated embryos, while we observed higher mortality rates at the supratherapeutic concentrations (15 and 20 µM) (mortality rates of 5.25%, 6.25%, 14.5% and 29.25%, respectively, at 1, 5, 10 and 20 µM concentrations) (Supplementary Figure S2A). Despite low mortality, we observed morphological deformities of alive embryos already at the lowest subtherapeutic 1 μM concentration (Supplementary Figure S2B). At this concentration, which we chose for all further experiments, we clearly distinguished malformed embryos with a mild or a severe phenotype (defined by the presence of at least one of the morphological defects described in Table 1). Developmental defects were already evident at 24 hpf, and especially for embryos with severe malformations, defects worsened at 48 hpf. Overall, at both 24 and 48 hpf, we observed 66% mildly and 29% severely affected embryos, i.e., with at least one of the mild or severe defects described in Table 1, respectively. Mostly, we observed shorter body length and curved tail, resulting in the disruption of the AP axis at both time endpoints (Supplementary Figure S2C). Pericardial edema was also evident in all severely affected embryos at 48 hpf (Supplementary Figure S2B), like in embryos exposed at 6 hpf.

**Figure 2:**
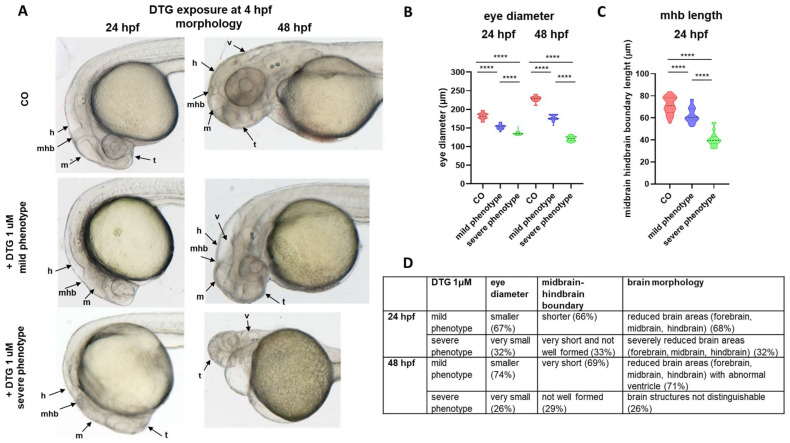
Brain morphology assessment of zebrafish embryos after exposure to dolutegravir (DTG) at 4 h post fertilization (hpf) (blastula stage). Dechorionated zebrafish embryos were exposed at 4 hpf (during the blastula stage) to drug solvent only (fish water plus 0.1% dimethyl sulfoxide, DMSO) (CO) or DTG 1 μM, dissolved in fish water containing 0.1% DMSO. (**A**) Representative pictures at 24 and 48 hpf of the gross morphological effects of 1 μM DTG exposure on brain development. Embryos were distinguished with mild or severe phenotype, based on the presence of at least one of the morphological defects described in Table 1. All treatments, including controls (CO), were conducted three times, with 30 embryos for each experiment and treatment (a total of 90 embryos for each experimental condition). Embryos in the figures are representative of all experiments. All pictures are lateral views with dorsal to the top and anterior to the left (magnification 40×) (h, hindbrain; m, midbrain; mhb, midbrain–hindbrain boundary; t, telencephalon; v, ventricle IV). (**B**) Violin plot distribution of eye diameter measurements at 24 and 48 hpf for control (CO) and 1 µM DTG-exposed embryos, distinguished as with mild or severe phenotype (x-axis). Measurements were performed on digital images using the ImageJ (Version 1.8.0) Software. The y-axis shows eye diameter measurements (μM). Measures were performed on 10 embryos for each of three experiments and for each treatment (a total of 30 embryos for each experimental condition). Inside each violin, the central dashed line marks the median (Q2) and the superior and inferior dotted lines mark the third (Q3) and first (Q1) quartiles. Eye diameter measurements were analyzed amongst and between groups by the 1-way ANOVA test with post hoc Tukey’s multiple comparison test (**** *p* < 0.0001). (**C**) Violin plot distribution of midbrain–hindbrain boundary (mhb) measurements at 24 hpf for control (CO) and 1 µM DTG-exposed embryos, distinguished as with mild or severe phenotype (x-axis). Measurements were performed on digital images using the ImageJ (Version 1.8.0) Software. The y-axis shows mhb length (μM). Measurements were performed on 10 embryos for each of three independent experiments and for each treatment (a total of 30 embryos for each experimental condition). Inside each violin, the central dashed line marks the median (Q2) and the superior and inferior dotted lines mark the third (Q3) and first (Q1) quartiles. Midbrain–hindbrain (mhb) measurements were analyzed amongst and between groups by the 1-way ANOVA test with post hoc Tukey’s multiple comparison test (**** *p* < 0.0001). (**D**) Table summarizing brain morphological defects at 24 and 48 hpf in 1 µM DTG-treated embryos, distinguished as with mild or severe phenotype, and showing % of embryos with each defect.

At the lowest subtherapeutic 1 μM DTG concentration, morphological deformities were also evident in the brain region, where we could again distinguish embryos with mild or severe phenotypes, at both time endpoints. Notably, we observed smaller eyes and reduction and malformation of most brain areas (forebrain, midbrain and hindbrain), with decreased length of the midbrain–hindbrain boundary at 24 hpf. Perturbed brain morphology worsened with time of exposure, and at 48 hpf, both mildly and severely affected embryos had smaller eyes, but also showed an abnormal and enlarged ventricle IV structure; further, in severely affected embryos, the main brain structures were not distinguishable and midbrain–hindbrain boundary seemed not formed or deformed (Figure 2A–D).

After exposure of embryos to 1 μM DTG, we further measured embryo mortality at a later stage (144 hpf) and compared it to embryo mortality at 48 hpf. As expected, also considering the observed morphological malformations, we found higher mortality at 144 hpf. However, most dead embryos were derived from those with a former severe phenotype (26% dead embryos), while embryos with a former mild phenotype mostly survived (16% dead embryos), although with morphological defects (Supplementary Figure S3).

Larvae treated with the lowest concentration of 1 μM DTG and with a mild phenotype were then selected in further experiments to further investigate defects that we observed in brain morphology.

### 2.2. Gross Morphological Malformations Induced by Early Embryonic Exposure of Zebrafish Embryos to Dolutegravir Are Prevented by Pre-Exposure and Rescued by Post-Exposure to Folic Acid

**Figure 3:**
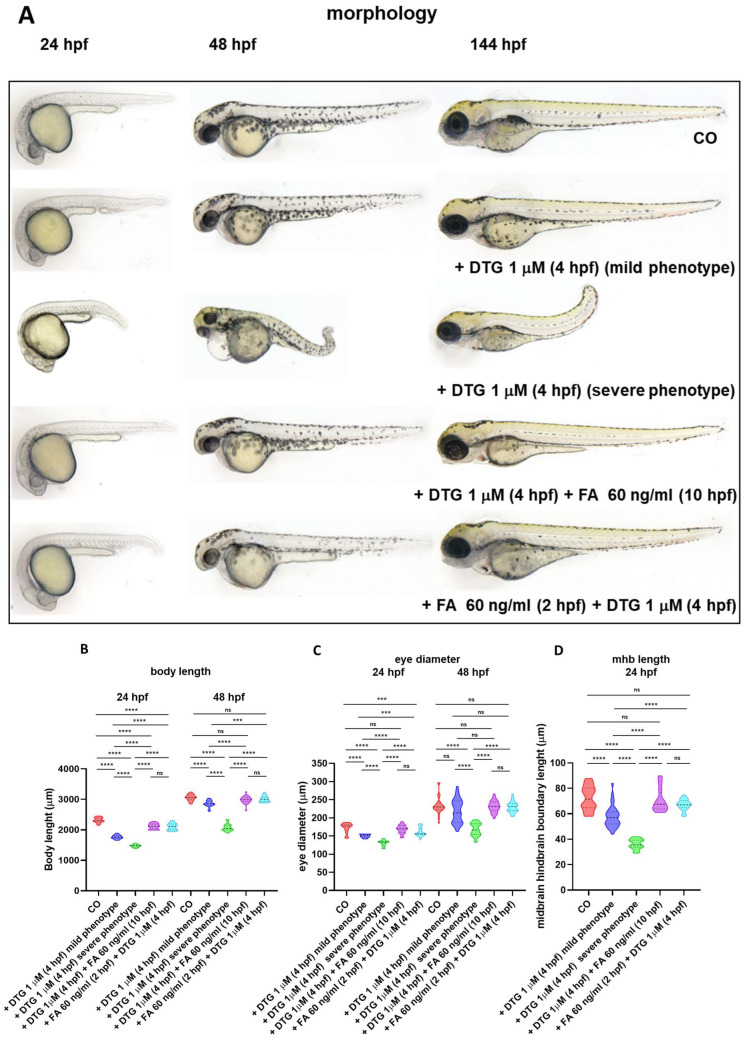
Folic acid (FA) both prevents and rescues gross morphological body and brain malformations induced by dolutegravir (DTG) exposure in zebrafish embryos at the blastula stage. Dechorionated zebrafish embryos were exposed to drug solvent only (fish water plus 0.1% dimethyl sulfoxide, DMSO) (CO); 1 μM DTG only (at 4 hpf, during the blastula stage); FA 60 ng/mL at 2 hpf (pre-exposure) plus 1 μM DTG at 4 hpf; or 1 μM DTG at 4 hpf plus FA 60 ng/mL at 10 hpf (post-exposure) (drugs dissolved in fish water containing 0.1% DMSO). (**A**) Representative pictures at 24, 48 and 144 hpf of the gross morphological effects of treatments, with DTG-treated embryos distinguished in embryos with mild or severe phenotype. All treatments were conducted three times, with 30 embryos for each experiment and treatment (a total of 90 embryos for each experimental condition). Embryos in the figures are representative of all experiments. All pictures are lateral views with dorsal to the top and anterior to the left (magnification 20×). (**B**) Violin plot of body length measurement at 24 and 48 hpf for control (CO), 1 µM DTG-exposed embryos, distinguished as with mild or severe phenotype (x-axis), and embryos treated with DTG and supplemented with FA by both post- and pre-exposure. The y-axis shows body length (μM). Measurements were performed on digital images using the ImageJ (Version 1.8.0) Software, considering the AP axis, on 10 embryos for each of three experiments and each treatment (a total of 30 embryos for each experimental condition). Inside each violin, the central dashed line marks the median (Q2) and the superior and inferior dotted lines mark the third (Q3) and first (Q1) quartiles. (**C**) Violin plot distribution of eye diameter measurements at 24 and 48 hpf for control (CO) and 1 µM DTG-exposed embryos, distinguished as with mild or severe phenotype (x-axis). Measurements were performed on digital images using the ImageJ (Version 1.8.0) software. The y-axis shows eye diameter measurements (μM). Measurements were performed on 10 embryos for each of three experiments and for each treatment (a total of 30 embryos for each experimental condition). Inside each violin, the central dashed line marks the median (Q2); the superior and inferior dotted lines mark the third (Q3) and first (Q1) quartiles. (**D**) Violin plot distribution of midbrain–hindbrain boundary (mhb) measurements at 24 hpf for control (CO) and 1 µM DTG-exposed embryos, distinguished as with mild or severe phenotype (x-axis). Measurements were performed on digital images using the ImageJ (Version 1.8.0). The y-axis shows mhb length (μM). Measurements were performed on 10 embryos for each of three independent experiments and for each treatment (a total of 30 embryos for each experimental condition). Inside each violin, the central dashed line marks the median (Q2); the superior and inferior dotted lines mark the third (Q3) and first (Q1) quartiles. Measurements were analyzed amongst and between groups by the 1-way ANOVA test with post hoc Tukey’s multiple comparison test (ns, statistically not significant; *** *p* < 0.001; **** *p* < 0.0001).

Since DTG developmental toxicity in zebrafish embryos is reduced by the supplementation with FA [10], we supplemented embryos with FA both before (2 hpf, immediately preceding the blastula stage starting) or after (10 hpf, at the end of gastrulation, when segmentation and organogenesis start) the exposure to DTG 1 μM (4 hpf) (Figure 1). We evaluated morphology at 24, 48 and 144 hpf, compared to control and 1 μM DTG-treated embryos. Most morphological body malformations were both prevented by FA pre-exposure and rescued by FA post-exposure, and interestingly, no evident gross morphological defects were observed in the brain region at all time endpoints (Figure 3A). Of note, in both conditions of FA supplementation, we did not observe embryos classifiable as with the severe phenotype, unlike in embryos treated with DTG only. We also measured body length, eye diameter and midbrain–hindbrain boundary length as for previous experiments (Figure 3B–D). While both FA pre-exposure and post-exposure did not prevent or rescue, respectively, the reduced body length at 24 hpf, conversely in both conditions of FA supplementation embryos were the same size as the control embryos at 48 hpf (Figure 3B). Likewise, eye diameter and midbrain–hindbrain boundary length of both FA pre-exposed and post-exposed embryos were not significantly different from those of control embryos, except for the eye diameter at 24 hpf for FA pre-exposed embryos, defect that was however recovered at 48 hpf (Figure 3C,D). These results suggested that at least gross morphological malformations in the body and developing brain regions induced by DTG exposure in zebrafish embryos are both prevented and rescued by FA supplementation.

### 2.3. DTG Exposure Induces Neurodevelopmental Defects over Time That Are Rescued by Post-Exposure Folate Supplementation

**Figure 4:**
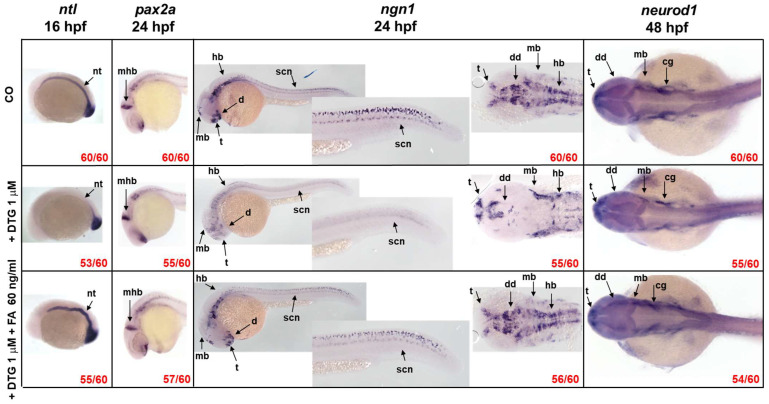
Neurodevelopmental defects induced overtime by dolutegravir (DTG) exposure of zebrafish embryos at the blastula stage are mostly rescued by post-exposure folic acid (FA) supplementation. Dechorionated zebrafish embryos were exposed to drug solvent only (fish water plus 0.1% dimethyl sulfoxide, DMSO) (CO) and 1 μM DTG only (at 4 hpf, during the blastula stage) or plus FA 60 ng/mL at 10 hpf (post-exposure) (drugs dissolved in fish water containing 0.1% DMSO). Transcript expression of *no tail (ntl)* (16 hpf), *paired box a (pax2a)*, *neurogenin 1 (ngn1)* (24 hpf) and *neuronal differentiation 1 (neurod1)* (48 hpf) genes was analyzed by whole-mount in situ hybridization (WISH). Representative images of three distinct performed experiments with 20 embryos for each experiment and each condition (total number of embryos with phenotype are shown in red numbers for each experimental condition). Lateral views with dorsal to the top and anterior to the left (magnification 20×) are shown for *ntl, pax2a* and *ngn1* genes. Further magnification (40×) was obtained for *ngn1* in the region of the spinal cord. Dorsal views are shown for *ngn1* and *neurod1* expression. Abbreviations: cg, cranial ganglia; d, diencephalon; dd, dorsal diencephalon; hb, hindbrain; mhb, midbrain–hindbrain boundary; mb, midbrain; nt, notochord; scn, spinal cord neurons; t, telencephalon.

We then performed experiments to further investigate the mechanisms of DTG effects on zebrafish neurodevelopment, performing all experiments after 1 μM DTG exposure (at 4 hpf), without FA supplementation or with FA given at 10 hpf (Figure 1), considering that the above data did not differ significatively between FA pre-exposure and post-exposure conditions. We chose to give FA at 10 hpf also considering that neurulation in humans begins two to three weeks after conception, the neural tube closes at the 4th week of pregnancy and neurogenesis starts at approximately gestational week 4 [17,18] (10 hpf in the development of zebrafish embryo corresponds to the end of gastrulation that is the end of the 3rd week of gestation in humans). We first used the WISH technique to study the mRNA expression of genes specifying transcription factors that are crucial in the neurodevelopment of zebrafish embryos (Figure 4).

Since NTDs were observed after in utero exposure to DTG, first of all, we analyzed the expression of the *ntl* gene. In comparison to control embryos, embryos exposed to DTG alone exhibited a dramatically reduced expression of *ntl* in the notochord region at 16 hpf, while maintaining a normal expression in the tail bud. FA supplementation after DTG exposure restored *ntl* expression in the notochord region, although the structure appeared malformed/deformed (Figure 4).

The expression pattern of the *pax2a* gene, observed by WISH at 24 hpf (Figure 4), confirmed our previous observation regarding the reduced length of the midbrain–hindbrain boundary after DTG exposure (Figure 2D), while FA supplementation allowed the maintenance of the boundary length. DTG-treated embryos also showed immature and smaller eyes, as already observed (Figure 2A,B), with mispatterned *pax2a* expression in this region, while DTG-treated embryos supplemented with FA exhibited a *pax2a* expression pattern in the eye region more similar to that of control embryos.

Then, we studied the expression of *ngn1*, a transcription factor expressed at the very onset of neurogenesis in the zebrafish neural plate. As observed in control embryos, at 24 hpf, *ngn1* is expressed in several regions of the brain, like the forebrain (telencephalon and anterior and posterior diencephalon), midbrain, hindbrain and along the spinal cord where both motor neurons and primary mechanosensory neurons reside. DTG exposure induced an evident downregulation of *ngn1* expression in all the main brain and spinal cord regions; furthermore, *ngn1* seemed misexpressed, as shown in the dorsal view of the embryos. Interestingly, DTG exposure also resulted in decreased *ngn1* expression in the region of the ventral and dorsal diencephalon where the earliest dopaminergic neurons are detected at 24 hpf in zebrafish embryos (Figure 4). The supplementation with FA rescued the correct expression pattern of the transcription factor, and the main brain structures seemed mostly restored (Figure 4).

**Figure 5:**
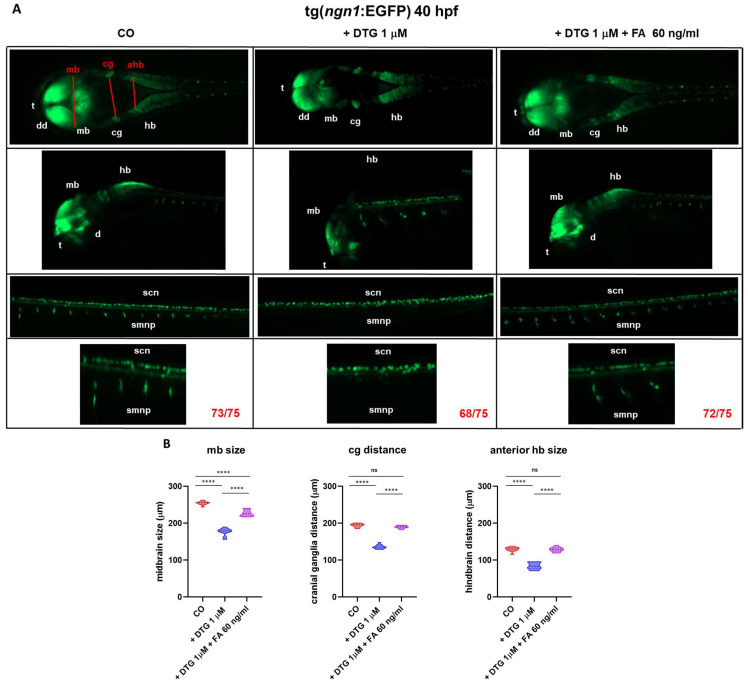
*ngn1*-dependent EGFP fluorescence in zebrafish embryos exposed to dolutegravir (DTG) at the blastula stage, with or without folic acid (FA) supplementation. Dechorionated zebrafish embryos [transgenic line Tg(*ngn1*:EGFP)] were exposed to drug solvent only (fish water plus 0.1% dimethyl sulfoxide, DMSO) (CO) and 1 μM DTG only (at 4 hpf, during the blastula stage) or plus FA 60 ng/mL at 10 hpf (post-exposure) (drugs dissolved in fish water containing 0.1% DMSO) and up to 40 hpf. (**A**) Representative images (obtained with fluorescence microscope) of three distinct performed experiments with 25 embryos for each experiment and each condition (total number of embryos with phenotype are shown in red numbers for each experimental condition). Dorsal and lateral views of the head region and of the spinal cord region (magnification 20×) are shown, with further magnification (40×), obtained for the region of the spinal cord, with magnification of the spinal cord neurons and spinal motor neuron projections. Abbreviations: d, diencephalon; dd, dorsal diencephalon; hb, hindbrain; mb, midbrain; scn, spinal cord neurons; smnp, spinal motor neuron projections; t, telencephalon. (**B**) In the dorsal view of Figure 5A, red lines and letters indicate midbrain size (mb), cranial ganglia distance (cg) and anterior hindbrain size (ahb). Measurements for the midbrain size (width), cranial ganglia distance (right-to-left cranial ganglia) and anterior hindbrain size (width) were analyzed using ImageJ (Version 1.8.0) Software, in three distinct experiments and in 10 embryos per treatment (a total of 30 embryos for each experimental condition). Data are presented as violin plots. Inside each violin, the central dashed line marks the median (Q2); the superior and inferior dotted lines mark the third (Q3) and first (Q1) quartiles. Statistical significance amongst and between groups was calculated by the 1-way ANOVA test with post hoc Tukey’s multiple comparison test (ns, statistically not significant; **** *p* < 0.0001).

To further confirm the effect of DTG on zebrafish neurogenesis and particularly on the expression of *ngn1* evaluated above by WISH, we then used a transgenic line expressing the Enhanced Green Fluorescent Protein (EGFP) under the control of the *ngn1* promoter, referred to as Tg(*ngn1*:EGFP). At 40 hpf, we observed a reduction in the areas expressing EGFP in the regions of telencephalon, diencephalon, midbrain and hindbrain, as also observed in WISH experiments at 24 hpf (Figure 5A). Compared to control embryos, we observed a significant decrease in midbrain width, cranial ganglia distance and anterior hindbrain width in DTG-treated embryos, while FA supplementation partially (midbrain width) or completely (cranial ganglia distance and anterior hindbrain width) rescued the phenotype (Figure 5B). Interestingly, we also observed that in DTG-treated embryos the EGFP signal was almost completely absent in the region of the spinal motor neuron projections, whose development is stimulated by brain dopaminergic neurons [19,20]. All defects seemed again reverted by FA supplementation (Figure 5A).

*neurod1* is another transcription factor involved in zebrafish embryo neurogenesis, which is induced by the transient expression of *ngn1*. DTG-exposed embryos exhibited a volume reduction in almost all of the dorsal brain areas, as already observed in the Tg(*ngn1*:EGFP) model. The supplementation with FA seemed again to rescue the correct expression of *neurod1,* particularly with volumes in the dorsal regions of the brain more comparable with those of control embryos (Figure 4).

Together, these results suggest that DTG exposure induces the dysregulation of crucial genes involved in embryo neurogenesis with profound neurodevelopmental defects over time that are mostly rescued by FA supplementation.

### 2.4. DTG Exposure Results in Decreased Hatching Rate, Locomotor Activity and Touch-Evoked Swimming That Are Rescued by Folate Supplementation

Since embryos treated with DTG showed a severe neurodevelopmental impairment, particularly in the brain dopaminergic regions, accompanied by decreased *ngn1* expression in spinal cord neurons and spinal motor neuron projections, we assessed the possibility that these disruptions may reflect in dysfunctional locomotor behaviors.

**Figure 6:**
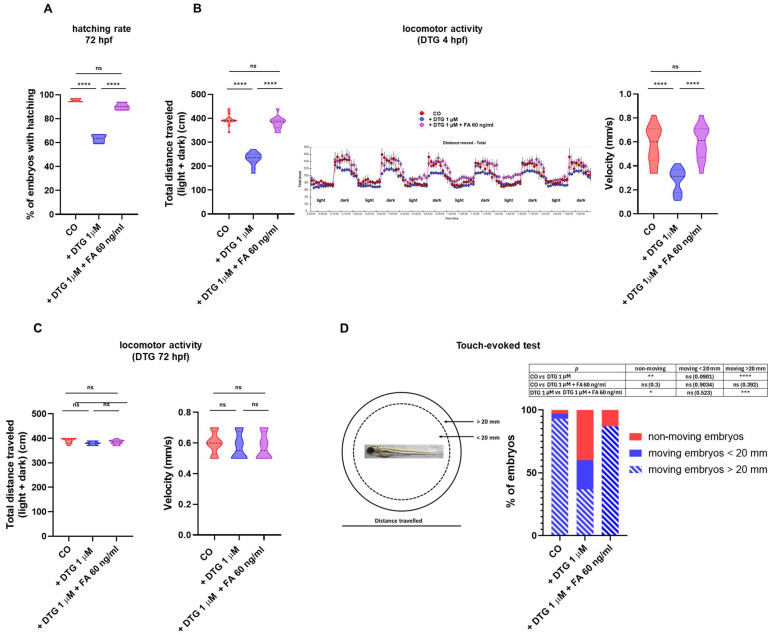
Hatching, locomotor activity and tactile-response deficit induced by dolutegravir (DTG) exposure in zebrafish embryos at the blastula stage are mostly rescued by post-exposure folic acid (FA) supplementation. Zebrafish embryos were exposed to drug solvent only (fish water plus 0.1% dimethyl sulfoxide, DMSO) (CO) for all experiments; 1 μM DTG only (at 4 hpf, during the blastula stage for hatching rate, locomotor activity and touch-evoked tests or at 72 hpf for locomotor activity tests only); or 1 μM DTG (at 4 hpf, for hatching rate, locomotor activity and touch-evoked tests or at 72 hpf for locomotor activity test only) plus FA 60 ng/mL given at 10 hpf (drugs dissolved in fish water containing 0.1% DMSO). Only for hatching experiments, non-dechorionated eggs were exposed, while dechorionated eggs were used, as for all previous experiments, for locomotor behavior and touch-evoked experiments. (**A**) Percentages of hatched embryos at 72 hpf. The x-axis shows treatments; the y-axis shows the corresponding percentages of hatched embryos at 72 hpf. All treatments, including controls (CO), were conducted three times, with 30 embryos for each experiment and treatment (a total of 90 embryos for each experimental condition). Results are expressed with violin plot distribution [inside each violin, the central dashed line marks the median (Q2); the superior and inferior dotted lines mark the third (Q3) and first (Q1) quartiles] (**B**). Embryos were exposed to treatments, considering DTG exposure at 4 hpf and FA post-exposure at 10 hpf. Locomotor activity was defined by total distance swam by the larvae (cm) and velocity (mm/s), calculated during both light and dark stimuli (moved distance in 2 min time bins, recorded for 2 h, 6 cycles of alternating light and dark 10 min periods). Measurements were performed with 12 embryos for each of three independent experiments and each treatment (a total of 36 embryos for each experimental condition). Results are expressed with violin plot distributions [inside each violin, the central dashed line marks the median (Q2); the superior and inferior dotted lines mark the third (Q3) and first (Q1) quartiles]. The raw data plot, representing mean ± SD of the distance moved by embryos in 2 min of time bins of one of three performed experiment evaluated by the Noldus *Ethovision* software (Version XT 13.0), is also shown. (**C**) Embryos were exposed to treatments, considering FA pre-exposure at 10 hpf and DTG exposure at 72 hpf. Locomotor activity was defined as above. Measurements were performed with 12 embryos for each of three independent experiments and each treatment (a total of 36 embryos for each experimental condition). Results are expressed with violin plot distributions [inside each violin, the central dashed line marks the median (Q2); the superior and inferior dotted lines mark the third (Q3) and first (Q1) quartiles]. (**D**) Embryos were exposed to treatments, considering DTG exposure at 4 hpf and FA post-exposure at 10 hpf. After gently touching the tail of each embryo, touch swimming response was observed and categorized as follows: embryos that did not move; embryos that swam < 20 mm within the motility wheel; embryos that swam > 20 mm in the wheel. Percentages of each category for each treatment were then calculated, considering 20 embryos for each treatment and each of three experiments, and plotted. Significance was analyzed by the 1-way ANOVA test with post hoc Tukey’s multiple comparison test (ns, statistically not significant; * *p* < 0.05; ** *p* < 0.005; *** *p* < 0.001; **** *p* < 0.0001).

Hatching is a physiological process in zebrafish embryos that occurs between 48 and 72 hpf and is strictly correlated to neurodevelopment and needs movements of the tail [21,22,23,24]. We then measured hatching rate after DTG exposure of non-dechorionated eggs from 4 hpf to 72 hpf, with or without FA supplementation given at 10 hpf. As shown in Figure 6A, DTG exposure of dechorionated eggs decreased their hatching rate, compared to control eggs, while FA supplementation restored a physiological rate.

To further assess the possibility of functional consequences of DTG exposure on locomotor activity of zebrafish embryos, we studied their swimming behavior after continuous drug exposure from 4 hpf to 144 hpf by a classic light–dark locomotion test. DTG severely reduced the total (light + dark) distance swam by the exposed larvae, which also displayed a decreased movement speed (Figure 6B). Again, FA supplementation at 10 hpf largely restored physiological movements, with total distance swam and movement speed similar to those of untreated larvae.

To determine whether DTG functional effects on larvae movements were strictly linked to the drug disrupting effects on the crucial phases of embryo neurodevelopment, we further studied their swimming behavior after drug exposure from 72 hpf to 144 hpf, considering that at 72 hpf neurogenesis is already completed. DTG exposure from 72 hpf did not reduce the total (light + dark) distance swam by the exposed larvae nor decreased movement speed (Figure 6C). FA supplementation at 10 hpf did not have further effects on locomotor behavior.

These data clearly showed that DTG exposure of zebrafish embryos during the most crucial phases of neurodevelopment results in a significant loss of motor performance at 144 hpf that may be completely rescued by FA supplementation, while drug exposure during a later phase of development (72 hpf), after completion of neurogenesis, has no effects on locomotor behavior.

In the spinal cord also reside the primary mechanosensory neurons of zebrafish embryo. To examine whether DTG exposure also affected the somatosensorial system, we performed a touch-evoked test by gently touch the tail with a pipette tip and recording the distance swam after the stimulus (< or >20 mm) at 72 hpf. Almost all (93%) control embryos were able to travel a distance > 20 mm, with only a little percentage of embryos swimming < 20 mm (4%) or not moving (3%). In contrast, among DTG-exposed embryos, 40% did not respond to the touch stimulus, 23% travelled a distance < 20 mm and only 37% displayed a regular touch response, travelling a distance > 20 mm. FA supplementation almost completely restored the touch-evoked behavior, with 87% of embryos swimming > 20 mm, although no embryos swam a distance < 20 mm and 13% of embryos displayed no movements (Figure 6D). These data suggested that DTG exposure, besides affecting spinal motor neuron functions, may also result in the impairment of the mechanosensory system of the spinal cord, further impacting the embryo locomotor behavior.

### 2.5. DTG Exposure Decreases the Number of Dopaminergic Neurons in the Dorsal Diencephalon While Folate Supplementation Rescued This Loss

**Figure 7:**
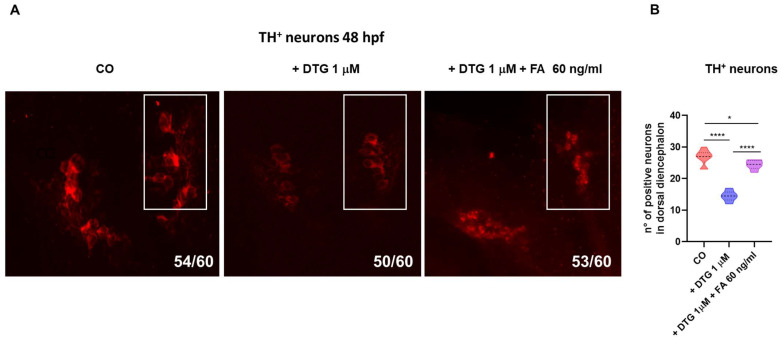
DTG exposure of zebrafish embryos decreases the number of tyrosine hydroxylase positive (TH^+^) dopaminergic neurons in dorsal diencephalon while folic acid (FA) supplementation partially rescues the deficit. Dechorionated zebrafish embryos were exposed to drug solvent only (fish water plus 0.1% dimethyl sulfoxide, DMSO) (CO) and 1 μM DTG only (at 4 hpf, during the blastula stage) or plus FA 60 ng/mL at 10 hpf (post-exposure) (drugs dissolved in fish water containing 0.1% DMSO). Immunofluorescence for TH was performed with rabbit anti-TH specific antibody at 48 hpf. (**A**) TH-stained embryos were visualized at 20× magnification with fluorescence microscope (dorsal view). All treatments, including controls (CO), were conducted three times, with 20 embryos for each experiment and treatment (a total of 60 embryos for each experimental condition). (**B**) Violin plot distribution of TH^+^ neurons. A defined area (white box) was analyzed to count the number of TH^+^ neurons, using Zen 3.5 (blue Version) software (Carl Zeiss AG). The x-axis shows treatments; y-axis shows the corresponding number of TH^+^ neurons counted in the defined area. Measurements were performed with 20 embryos for each of three experiments and for each treatment. Inside each violin, the central dashed line marks the median (Q2); the superior and inferior dotted lines mark the third (Q3) and first (Q1) quartiles. Significance was analyzed by the 1-way ANOVA test with post hoc Tukey’s multiple comparison test (* *p* < 0.05; **** *p* < 0.0001).

Descending dopaminergic projections from the brain promote motor neuron generation in the developing zebrafish [20]. Since we observed the reduced expression of *ngn1* in the dopaminergic regions of the forebrain, spinal cord and trunk motor neuron projections and decreased hatching rate and locomotor activity of embryos exposed to DTG, with the partial or complete rescue of all those defects by FA supplementation, we examined brain dopaminergic tyrosine hydroxylase positive (TH^+^) neurons by immunohistochemistry in DTG-treated embryos, in comparison with control and FA-supplemented embryos. The analysis showed that the number of TH^+^ cells was decreased in DTG-exposed in comparison with those in control larvae, while FA-supplemented embryos showed a number of TH^+^ neurons more similar to that in untreated ones, although still significantly different in comparison with that in control embryos (Figure 7A,B).

## 3. Discussion

In the present study, we ascertained the neurodevelopmental toxicity of DTG and the effect of FA supplementation during zebrafish embryogenesis. After early embryonic exposure to a subtherapeutic DTG concentration, we observed several morphological malformations, mainly in the developing nervous system, ranging from mild to severe and worsening over time of exposure. Particularly, we observed morphological defects in the forebrain, midbrain and hindbrain regions, with a reduced size of the midbrain–hindbrain boundary and the eye region and the disruption of the AP axis. At the molecular level, we found that DTG exposure impaired expression of genes expressing transcription factors that are crucial during the early phases of neurodevelopment. Of note, we observed a decreased and disrupted expression of the *ngn1* transcription factor in the dopaminergic regions of the developing forebrain, in the spinal cord neurons and spinal motor neuron projections of the trunk, accompanied by the depletion of TH^+^ dopaminergic neurons of the dorsal diencephalon and the reduction in larvae locomotion in response to DTG exposure even at subtherapeutic concentration. These abnormalities were rescued, although not completely, by FA supplementation. Our study then further supports previous evidence that DTG can interfere with FA pathways and also provides new insights regarding the mechanisms involved in the increased risk of DTG-associated fetal neurodevelopmental defects and adverse neurologic outcomes in children exposed in utero to DTG [8,10,11,12,14,18,25,26,27,28].

The potential teratogenic effect of DTG during pregnancy is still a matter of debate; although some studies reported a higher rate of newborns with NTDs, low folate intake in some populations may explain those findings [4,5]. DTG regimens also result in neuropsychiatric symptoms, like anxiety, depression and suicidal behavior [1,3], and an increased risk of DTG-related neurological damage has recently been observed in HIV-uninfected children, but HIV/DTG-exposed during their in utero life [8].

Zebrafish embryos are widely used to study drug neurotoxicity during neurodevelopment, thanks to many advantages, notably its transparency, external and fast development of eggs and the possibility to easily monitor changes in locomotor behavior [15,16]. In this study, we firstly observed that survival and morphology of embryos were affected by DTG exposure when exposure begun during the blastula stage (4 hpf), while exposure during gastrulation (6 hpf) resulted in lower toxicity. These observations are in accordance with the previous ones obtained in this animal model, suggesting a critical period of exposure during pregnancy to explain DTG teratogenicity [10]. Our findings also expand previous observations of Cabrera and colleagues [10] that were obtained with very high concentrations of DTG (100 μM, that is 10× the human C_max_), since we observed a significant embryo mortality rate even at the subtherapeutic concentration of 1 μM (0.1× the human C_max_). The effects on embryo survival were evident at early stages (24 and 48 hpf) and worsened at later larval stages (144 hpf). These observations could explain the recently described higher risk of pregnancy loss after periconceptional or early-pregnancy DTG exposure [7].

DTG pharmacokinetics is different in humans and zebrafish, and we cannot “*a priori*” predict the embryotoxic dose for zebrafish embryos, based on studies in humans or other mammalian species. Embryotoxicity studies in murine pregnancy models [26,29] suggested that DTG peak plasma levels of about 3000 ng/mL (corresponding to 6.8 μM) may be considered as 1× therapeutic plasma level in mice. This dose was associated with increased rates of fetal defects in mice. To the best of our knowledge, the only experimental evidence of DTG toxicity in zebrafish embryos has been described by Cabrera and colleagues [10], using a very high drug dose (10× the human C_max_, 100 μM), but the researchers observed a high developmental toxicity (80–100%) at 24 hpf that was however rescued by co-exposure with FA. The zebrafish embryo model is intensively studied as a developmental toxicity model for predicting embryotoxicity of drugs and chemicals, and recent studies have demonstrated that zebrafish embryo development assays can predict, within 1-log, the rat maternal exposure levels associated with drug or chemical embryotoxicity 75% of the time [30]. Then, we may realistically suppose that the 1 μM dose used in this study should be considered a subtherapeutic dose.

Of great importance in this study is that, after early exposure of embryos to 1 μM DTG, we observed not only the disruption of the AP axis, suggestive of NTDs, but also gross anatomical defects in the main brain regions that led us to further investigate the underlying mechanisms. We particularly observed malformations and reduced areas of the developing forebrain, midbrain and hindbrain regions, smaller eyes and decreased length of the midbrain–hindbrain boundary. Gross morphological defects, particularly evident in a significant percentage of severely affected embryos (29%), worsened between 24 and 48 hpf and led to a high mortality rate in these more severely affected embryos in later stages (144 hpf).

The midbrain–hindbrain boundary represents a crucial constriction region in the developing brain that functions in morphologically defining distinct brain regions, the rostral and caudal territories of the midbrain and hindbrain, and in physically separating distinct ventricular regions. Importantly, in acting as an organizer region and through the expression of transcription factors and soluble signaling molecules, the boundary guides the fate of neighboring cells, determining neuronal migration and axonal guidance both anteriorly in the midbrain and posteriorly in the hindbrain and polarizing rostrally the dorsal mesencephalic regions involved in processing visual and acoustic sensory information and caudally the cerebellum, an important center involved in motor control [31]. The impaired development of the midbrain–hindbrain boundary in our model, observed both morphologically and quantified by measurement, was confirmed by the expression pattern of the transcription factor *pax2a* observed by WISH and is consistent with the morphological disruption of the midbrain, hindbrain and ventricle regions that we found in DTG-treated embryos in this study. The *pax2a* gene is indeed a transcription factor involved in the development and function of the midbrain and midbrain–hindbrain boundary, also affecting the development of the neighboring forebrain and hindbrain regions [32].

Consistent with previous findings [10], in the present study, we found that DTG-induced zebrafish embryo toxicity may be both prevented and rescued by FA supplementation in the early developmental phases. Survival rate was ameliorated and no gross morphological body deformities and, more interestingly, no gross brain malformations were found in both FA supplementation conditions in our model. All these findings are also coherent with the numerous observations in animal models and humans of a relationship among DTG exposure, sub-optimal folate levels and fetal defects, although the underlying mechanism of these evidences remain already mostly unclear [4,6,7,12,27]. Tukeman and colleagues [27] evaluated DTG developmental toxicity in fetuses from DTG-treated dams fed normal or low FA diet, finding NTD-affected fetuses (exencephaly) only with low FA diet. In a similar animal model, Mohan and colleagues [12,26] observed a higher rate of NTDs (exencephaly, encephalocele and spina bifida) and tail defects under folate-deficiency conditions, also together with severe left–right asymmetry, abdominal wall, limb and craniofacial anomalies, bleeding defects and severe edema. In our model, we as well observed the disruption of the AP axis and a strong reduction in *ntl* expression in the notochord after DTG exposure, both suggestive of NTDs, and the defects were rescued by FA supplementation, in agreement with what was previously observed in the murine model. More interestingly, only few studies have considered other aspects of DTG neurotoxicity, as we did in this work. Recently, several brain metabolites, particularly those related to pathways involved in energy metabolism and oxidative stress, have been found dysregulated in brain subregions of DTG-treated adult mice [33]. Further, proteomic profiling of brain tissues of murine fetuses derived from DTG-treated female mice revealed impairment in neurogenesis, synaptogenesis and neurite formation, while long-acting DTG nanoformulations given by injection mostly prevent those defects [34]. In utero DTG exposure has also been shown to inhibit fetal brain metalloproteinase activity, resulting in impaired post-natal neurodevelopmental defect in mice, suggestive of axonal and/or myelin damage, neuronal and/or synaptic damage and neuroinflammation, and in modified transcription of several genes involved in axonal guidance, synaptogenesis and neuroinflammation [14]. DTG has been found to induce the dysregulation of genes involved in early differentiation in human embryonic stem cells at a subtherapeutic dose (0.5× the human C_max_) and to decrease viability, neurite and synapse formation in neurons derived from human induced pluripotent stem cell [35]. In any of these studies, however, the researchers consider the possibility that FA supplementation may prevent or revert DTG neurotoxic effects. In this regard, our work adds something new to the puzzle of DTG neurotoxicity, since we considered this possibility, beyond the already known effects of FA on NTD and DTG-induced defects.

FA is a micronutrient, and its deficiency during pregnancy is indeed associated with an increased risk of NTDs in the fetus; low levels of folate in adults also seems to be related with depression and other cognitive disorders [36,37], and FA supplementation can improve both conditions in humans. While DTG seems to interfere with FA metabolism [10], on the other hand, a previous low level of FA during DTG intake could further reduce FA levels, inducing defects during neurogenesis in embryos and perhaps neuropsychiatric disorders in adults. In this regard, it would be of interest studying the cognitive performance in relation with FA levels in both adults on DTG and in children exposed in utero to the drug.

To further investigate the mechanisms of DTG effects on zebrafish neurodevelopment, we analyzed the expression of genes that are crucial in the earlier phases of CNS formation. We also evaluated the effects of FA supplementation given post-DTG exposure (10 hpf), considering that this moment, the end of gastrulation, precedes neurogenesis [17,18]. Embryonic neurodevelopment is orchestrated and coordinated in a finely controlled temporal and spatial order by several transcription factors [38,39,40,41]. The notochord is an early embryonic structure, which is the source of important signals for axis formation, somite patterning and, importantly, for the induction of the neural tube. The zebrafish *ntl* gene is the ortholog of the mammal *T-box transcription factor* (*TBXT*, *T Brachyury Transcription Factor*) and, as an early transcription factor, is required for mesoderm formation and differentiation, cell movements during gastrulation, notochord, posterior somite and tail development and for the establishment of the left–right symmetry [42,43,44,45]. Zebrafish *ntl* null mutants indeed lack notochord and posterior tail tissues [46]. The expression of *ntl* during embryo development is epigenetically determined by regulated changes of DNA methylation [47]. FA insufficiency has been shown to suppress *Brachyury* expression through DNA hypermethylation modification, inducing the disruption of fibroblast growth factor (FGF) pathways in humans and mice and in turn determining defects in neurological development, like NTDs [48]. Therefore, we analyzed *ntl* expression in an early embryonic developmental stage (16 hpf); accordingly, we found a strong reduction in *ntl* expression in the notochord after DTG exposure, which was rescued by FA supplementation. However, FA supplementation given at 10 hpf (the end of gastrulation in zebrafish, corresponding to the end of the 3rd week of gestation in humans) was not sufficient to form a morphologically correct notochord structure, since this appeared clearly distorted. These results can be explained considering that the notochord begins to appear just at the end of gastrulation in zebrafish embryos [38]; then, we can postulate that endogenous FA could only partially support *ntl* epigenetic activation and induction of *ntl*-controlled pathways in the presence of DTG. All nutrients needed for zebrafish embryo development are present in the yolk, and all genes involved in the folate cellular metabolic pathways can be found in embryos already at the 1- to 2-cell stage, due to the presence of maternally loaded transcripts that are expressed throughout early developmental phases [49]. DTG exposure could however induce a precocious although maybe partial FA deficiency within the time frame of 6 h (from 4 hpf, when DTG exposure starts, up to 10 hpf, when FA was supplemented) that seemed however sufficient to partially influence *ntl* expression and disrupt the notochord architecture. This observation further highlights the importance of FA supplementation in all women of child-bearing age under DTG therapy, even in FA-sufficient conditions.

The analysis of *pax2a* mRNA expression pattern confirmed the reduction in the length of the midbrain–hindbrain boundary, but also suggested eye defects. *pax2a* is critical for the embryonic eye development, in that its expression in the transitory optic stalk is involved in delineating the subsequent retinal axon outgrowth within the diencephalon [50]. The optic stalk is indeed a transient structure during the eye development that progressively decreases in size and is completely replaced by the optic nerve at 3 days post fertilization in zebrafish embryos. In *pax2a* null mutant zebrafish embryos, the stalk structure is retained, the choroid fissure fails to close and optic axons show abnormal trajectories. DTG-treated embryos showed smaller eyes and the development of the optic stalk seemed immature in comparison with both untreated and DTG-treated and FA-supplemented embryos, suggesting an improper or delayed eye development that may result in eye defects. Our observation in this model could also explain the eye defects recently found in murine fetuses after maternal exposure to the drug [26].

A further crucial gene in early embryonic neurodevelopment and neuronal determination is *ngn1*, a neural specific basic helix-loop-helix (bHLH) transcription factor, expressed at the very onset of neurogenesis in the zebrafish embryo neural plate domains that give rise to sensory neurons of the dorsal root ganglia and primary motor neurons that control embryo motility. *ngn1* is expressed prior to neural lineage determination and is required for the specification of the dopaminergic progenitor cells of the basal forebrain and is sufficient for the appearance of TH^+^ cells in those regions [51,52,53]. In our model, DTG exposure resulted in a severely compromised expression of *ngn1* in almost all the main regions of the developing CNS at 24 hpf, suggesting a profound effect of the drug on neurogenesis and neuronal differentiation. Areas expressing *ngn1* were also reduced in volume, as observed in dorsal view in the transgenic Tg(*ngn1*:EGFP) line at 40 hpf, and the reduced size of most brain regions were confirmed by WISH analysis for *neurod1* expression at 48 hpf, expected to be mainly present in differentiated neurons of the midbrain, hindbrain, midbrain–hindbrain boundary and cranio-facial ganglia [54,55]. Importantly, *ngn1* expression was severely decreased in areas of the nervous system that are crucial in regulating the locomotor pattern of zebrafish, in particular the dopaminergic regions of the forebrain, the spinal cord neurons and the spinal motor neuron projections, whose development is induced by the descending diencephalon–spinal dopaminergic projections themselves [20]. Parallelly, we observed a decreased number of TH^+^ neurons in the dorsal diencephalon of treated embryos at 48 hpf, confirming that the dopaminergic pathways were compromised by DTG exposure. FA supplementation after DTG exposure was sufficient to almost completely restore the correct expression of *ngn1*; it mostly preserved the size of all brain regions and also partially rescued TH expression in the dorsal diencephalon with a number of TH^+^ neurons nearest to those of untreated embryos. The disrupting effects on the diencephalic regions and spinal cord neurons were interestingly accompanied by dysfunctional hatching at 72 hpf and decreased locomotor activity at later stages, both completely rescued by FA supplementation. The dopaminergic system has several functions in mammals, being involved in motor activity, perception, behavior, emotions, mood, learning and attention. In humans, alterations in the dopaminergic pathways and projections are implicated in many neuropsychiatric disorders such as schizophrenia, attention-deficit/hyperactivity disorder, depression and addiction and in neurodegenerative diseases like Parkinson’s disease (PD). The dopaminergic system of zebrafish is not completely overlapping with that of mammals. Most significantly, the zebrafish lacks dopaminergic neurons in the midbrain; however, anatomical and functional correlations of dopaminergic system projections between mammalian and zebrafish have been described [56]. Importantly, zebrafish conserves the dopaminergic diencephalon–spinal tract, which provides spinal dopamine and is involved in locomotor development [19,56,57]. Dopaminergic TH^+^ neurons are detected early (24 hpf) in the zebrafish forebrain, and within 48 hpf, dopamine is released in the trunk and tail region close to progenitor cells of motor neurons, promoting their maturation and motor neuron development [20]. This dopaminergic signal is critical for zebrafish locomotor behavior, since an early reduction in dopamine signaling results in a reduced number of developing motor neurons that is not compensated for later in the development, resulting in reduced spontaneous swimming and vital motor behaviors at later stages [20]. Considering all the above observations in zebrafish embryo models and what we similarly observed in this study after DTG exposure, it can be speculated that DTG may act by altering the developing dopaminergic neurons of the forebrain and, as a consequence, reducing dopamine signaling in the spinal tract, in turn decreasing the development of functional spinal motor neurons and finally lowering locomotor activity at later stages. FA supplementation at 10 hpf, acting before the complete development of the dopaminergic system and the diencephalon–spinal tract may at least partially rescue those deleterious effects, also reducing their consequences on locomotor behavior. Of note, DTG exposure with FA supplementation at later stages of development (72 hpf), when the dopaminergic projections are already established in the trunk and tail and dopamine release in these regions has already initiated [20], did not impact the larval swimming behavior at later stages. It is tempting to speculate that FA could act in an epigenetic manner, altering the methylation pattern of important transcription factors such as *ngn1* and downregulating their expression, in a way similar to what was previously observed for Brachyury gene. Otherwise, FA might act on dopamine synthesis. Interestingly, folate-deprived adult zebrafish showed reduced swimming velocity accompanied by reduced dopamine production [58]. Then, our findings for the first time suggest the possible interaction of DTG with dopaminergic neuron development and/or dopamine metabolism that could explain neurologic dysfunctions in in utero exposed children [8] as well as the neuropsychiatric adverse events observed in adults treated with DTG [1,3]. Dopamine metabolism is strictly related to one-carbon metabolism, dopamine being degraded by catechol-*O* methyl transferase (COMT), which catalyzes the transfer of a methyl group from S-adenosylmethionine (SAM) to a hydroxyl group of dopamine or its derivative 3,4-dihydroxyphenylacetic acid, in turn producing homocysteine from methionine within the methionine cycle. The methionine cycle is interconnected with folate metabolism and depends on folate levels, in that methionine synthase, the enzyme that converts homocysteine to methionine in the cycle, uses 5-methyl-tetrahydrofolate (5-m-THF), an intracellular derivative of folate, as the substrate and vitamin B12 as a cofactor. Low levels of folate result in low levels of 5-m-THF, inhibiting the reconversion of homocysteine to methionine and resulting in high homocysteine levels. Increased levels of homocysteine due to deficiencies in one-carbon metabolism, related to both genetics and environmental factors like nutrition deficits, have been related to PD onset and progression, although the involved mechanisms are still unclear [59,60]. Noteworthy, maybe through the elevation of plasma homocysteine levels, is that dietary folate deficiency sensitizes dopaminergic neurons to dysfunction and death in a 1-methyl-4-phenyl-1,2,3,6-tetrahydropyridine (MPTP)-induced PD murine model, inducing severe motor dysfunctions [61], and homocysteine administration into the brain decreased the locomotor activities and dopamine levels, in the rat striatal regions [62]. Another possible mechanism through which folate deficiency might influence dopamine metabolism is by means of epigenetic mechanisms, impacting through modified DNA methylation levels the expression of genes involved in dopamine metabolism, such as TH that catalyzes the first step in dopamine synthesis [59,60]. TH activity is also strictly regulated on several other levels, strictly interconnected with folate metabolism. The active enzyme consists of four identical catalytically active subunits, each of them requiring tetrahydrobiopterin (BH4), ferrous ion and O_2_ to oxidize tyrosine to dopamine. Folate and biopterin coenzymes have structural and functional similarities, with metabolic interactions occurring between their pathways. BH4 cellular bioavailability is indeed derived from de novo synthesis starting from guanidine triphosphate (GTP) and guided by guanidine triphosphate cyclohydrolase (GTPCH), a magnesium, zinc and NADPH-dependent enzyme, or alternatively from the salvage pathway that regenerates BH4 from the BH2 oxidized form through the enzymatic activity of dihydrofolate reductase, involved in the folate metabolism [63,64]. Then, folate enhances regeneration of BH4 from the inactive form BH2, participating in the maintenance of adequate levels of BH4 for TH activity and dopamine synthesis.

As stated above, *ngn1* is also required for the development of sensory neurons of the zebrafish trunk, and it is expressed early in Rohon-Beard spinal sensory neurons, located within the spinal cord, and later in the dorsal root ganglia [65]. Indeed, the knock-down of *ngn1* impairs the formation of Rohon-Beard sensory neurons and dorsal root ganglia [66]. These sensory neurons are considered as touch-responsive neurons that induce locomotion following a tactile stimulus, as an escape behavior [66]. After DTG exposure, *ngn1* expression is lowered in the spinal cord neurons, where also primitive sensory neurons reside. The touch-evoked locomotor response of DTG embryos was reduced, while FA supplementation rescued, although not completely, the tactile swimming response. Our observation suggests that DTG exposure might also affect the somatosensorial system, although the reduced locomotion may also be caused by the impairment of dopaminergic and motor neurons described above.

Recently, data from mouse pregnancy models showed that folate deficiency increases the incidence of DTG-associated fetal defects in tail, abdominal wall, limb and craniofacial structures with frequent bleeds [12], while normal FA levels during pregnancy ameliorate developmental defects that arise from DTG exposure, especially NTDs [27]. In concordance, in countries where maternal food is supplemented with FA, the risk of DTG-associated fetal malformations, including NTDs, is lower than those reported from the initial surveillance data in Botswana where there are no FA fortification public programs [4,7]. All these studies suggest a potential relationship among DTG, sub-optimal folate levels and fetal defects. Mohan and colleagues [12] hypothesized that DTG might modify folate metabolism through a metabolic reaction named “methyl folate trap”, a metabolic consequence of vitamin B12 deficiency that causes functional folate deficiency [67]. Folate uptake into cells is followed by its conversion into 5,10-methylene-tetrahydrofolate (THF) that contributes, donating one carbon atom, to both thymidylate synthesis and to the methionine cycle, necessary to convert, through the activity of methionine synthase, the amino acid homocysteine into the amino acid methionine, in turn converted into SAM. Vitamin B12 deficiency indeed causes the inactivation of methionine synthase, irreversibly trapping folates into the 5-m-THF form at the expense of 5,10-methylene-THF and THF that are crucial for DNA synthesis and ultimately blocking it. The alteration of these methylation pathways also causes increased homocysteine levels and oxidative stress, further contributing to genomic instability. Of note, as reported above, increased levels of homocysteine may also act on dopamine production. The hypothesis of Mohan and colleagues [12] was based on the known property of DTG for zinc chelation and the need for zinc as a cofactor, together with vitamin B12, for the methionine synthase activity, suggesting the need of an additional dietary factor like vitamin B12 and zinc, besides FA, in the context of DTG use during pregnancy and potentially in all patients on DTG. Zinc has been already described as having an important role in regulating neuronal stem cell proliferation, survival and differentiation. Its deficiency may have implications for both developmental and adult neurogenesis [68], regardless of its effects on one-carbon and folate pathways, and this may be of importance, considering that in our neurodevelopmental model, FA does not completely rescue the normal phenotype. Studies in murine models have revealed that Zic3, a member of the zinc finger protein family, is critical for right–left differentiation. Zic3 null mice showed early gastrulation defects, with axial patterning defects and NTDs [69]. Interestingly, Zic3 is also an important regulator of proneural genes in zebrafish embryos, like *ngn1* and *neurod1* [70]. Further, due to their zinc-chelating activity, DTG and other INSTIs like bictegravir and cabotegravir have been shown to inhibit the enzymatic activity of the matrix metalloproteinase MMP-2 and MMP9 that play a crucial role in many neurodevelopmental processes, suggesting a further potential mechanism by which DTG could affect fetal neural development [14,18]. Of note, maternal zinc deficiency during pregnancy and lactation can affect learning and memory of offspring, probably through the modulation of proteins involved in DNA methylation and then in epigenetic control of genes related to cognitive functions [71]. Then, the chelating activity of DTG might be an important mechanism through which the drug could exert its neurodevelopmental and neurological effects.

DTG-fetal defects under folate-deficient conditions are not limited to NTDs. Mohan and colleagues [12] also described other fetal defects in mice including facial dysmorphology, severe left–right asymmetry phenotypes, abdominal wall and limb defects, bleeding and severe edema. Some of those defects may be explained by the altered expression profiles of developmental regulator genes involved in embryonic patterning and early differentiation induced by DTG exposure independent of FA deficiency, as recently suggested in in vitro studies [35,72]. Further, notochord is essential for the neural and endodermal patterning of, e.g., the intestine, liver and lungs, and in vertebrates, signaling molecules such as the hedgehog proteins secreted by the notochord play key roles in the differentiation and growth of the surrounding tissues [73,74]. Then, the effect of DTG exposure on notochord formation that we and others observed may reflect further embryonic defects, independent of NTDs.

Our study in zebrafish embryos has several advantages. First, zebrafish embryo itself, as a model of neurodevelopmental disorders and neurotoxicity, has the important advantages of external and fast development and of its transparency, making its CNS easily visible and accessible to morphological and molecular studies. Second, our study has the advantage of having observed anatomical defects, gene expression modulation and locomotor impairments caused by DTG and the effect of FA supplementation in a chronological way, studying every crucial step of neurodevelopment from the first phases of neurogenesis up to the complete development of mature swimming ability. Third, in this study, we have observed the effects of DTG only, without the possible confounding effects of further antiviral drugs. Fourth, here, we have described for the first time the effects of DTG exposure in embryos on locomotor activity and on the development of the dopaminergic system and dopaminergic projections and their impact on locomotor behavior development. Taken together, our findings then further support previous evidence that DTG can interfere with FA pathways, but also provide new insights regarding the mechanisms involved in the increased risk of DTG-associated fetal neurodevelopmental defects and adverse neurologic outcomes in children exposed in utero to DTG, suggesting the dopaminergic system as involved [8,10,11,12,14,18,25,26,27,28]. This study has also several limitations. First of all is the use of a non-mammalian model that shares with mammals many similarities in brain development, morphology, structure and function but also some diversities. Secondly, for most experiments, we supplemented embryos with FA at 10 hpf, that is 6 h after DTG exposure, considering that both pre- and post-supplementation resulted in no gross morphological defects, particularly in the brain, and produced no embryos with a severe phenotype in both cases; that this moment, the end of gastrulation, precedes neurogenesis [17,18]; and finally that the end of gastrulation corresponds to the end of the 3rd week of gestation in humans, approximately the moment a woman notices that she is pregnant. A consequence of this choice is possibly the non-complete rescue of some defects induced by DTG exposure. Considering that in many cases FA is usually given to women at the time they realize that they are pregnant (corresponding to about the end of the 3rd week of gestation in humans and about 10 hpf in zebrafish embryos), our observations however further highlight the importance of FA supplementation in all women of child-bearing age under DTG therapy, even in FA-sufficient conditions.

## 4. Materials and Methods

### 4.1. Ethics Statement

All experiments took place at the Zebrafish Facility, Department of Molecular and Translational Medicine, University of Brescia, Italy. All experiments were conducted in accordance with the Italian and European regulations on animal care and the standard rules defined by the Local Committee for Animal Health (Organismo per il Benessere Animale) and authorized by the Italian Ministry of Health (Authorization Number 585/2018).

### 4.2. Fish Maintenance and Collection of Eggs

Zebrafish embryos were obtained, as described below, by mating the AB wild-type line for all experiments, with the exception of those experiments described in Section 4.6, for which we used the transgenic line Tg(*ngn1*:EGFP). In the transgenic line, the zebrafish neural specific basic helix-loop-helix (bHLH) transcription factor *neurogenin 1 (ngn1)* promoter drives the expression of EGFP. *ngn1* is expressed in neuronal precursors at the very onset of neurogenesis in the zebrafish neural plate prior to neural lineage determination, and it is crucial for cellular differentiation in primary neurons that control embryo motility, dopaminergic neuron progenitors of the forebrain and progenitors of sensory neurons of the dorsal root ganglia [52]. Fish were bred in a recirculating aquaculture system (Techniplast ZebTEC, Buguggiate, Italy) in fish water (0.1 g/L Instant Ocean Sea Salts, 0.1 g/L sodium bicarbonate, 0.19 g/L calcium sulfate) at 28.5 °C in a 14 h light and 10 h dark daily cycle, essentially as previously described [75]. The fish were fed three times per day with dry granular feed (GEMMA Micro Skretting, Tooele, UT, USA) and one time per day with fresh Artemia. Adult male and female fish were mated in the breeding box overnight. Freshly spawned eggs were collected the next morning, washed and maintained at 28 °C in Petri dishes containing fresh fish water until 2 h post fertilization (hpf) (nearby the start of blastula stage, 2 and 1/4 hpf), 4 hpf (during the blastula stage), 6 hpf (nearby the onset of gastrulation, 5 and 1/4 hpf) or 10 hpf (the end of gastrulation), depending on the different experiments. Embryo staging was done as described by Kimmel and colleagues [76]. Embryos were finally exposed to the drugs at the indicated times for each experiment. The experimental exposure and analysis timelines are described below and summarized in Figure 1.

### 4.3. Drug Exposure of Embryos

A 1 mM (441 μg/mL) stock solution of DTG was first prepared by dissolving the drug (dolutegravir sodium, TA9491598175, Sigma-Aldrich, Saint Louis, MO, USA) in distilled water plus dimethyl sulfoxide (DMSO, 10% final concentration) (Sigma-Aldrich). This stock solution was used in our first pilot experiments to freshly prepare final exposure solutions for each tested concentration (obtained by serial dilutions of the stock in fish water). Since we noticed that DTG was not completely soluble in distilled water plus 10% DMSO and sometimes a visible particulate was evident, we sonicated the stock solution (two brief cycles of sonication, 15 s each) to obtain a clear solution with no visible particulate. We stored the 1 mM stock solution at 4 °C and sonicated it as above before each experiment, when a particulate was visible. No particulate was evident after serial dilutions of the stock in fish water (DTG sodium salt, water solubility 269 μg/mL) [77]. Nonetheless, to avoid the sonication step, in all the here-described experiments, we preferred to use a 10 mM (4.41 mg/mL) stock solution of DTG directly dissolved in 100% DMSO (no particulate was visible) and diluted this stock in fish water at the tested concentrations. A 60 µg/mL stock solution of folic acid (FA) (folic acid F8758, Sigma-Aldrich) was prepared by dissolving the drug in distilled water. All exposure solutions were freshly prepared for each tested concentration by serial dilutions of the stock 10 mM DTG or/and 60 µg/mL FA solutions in fish water. As the negative control and for 1 and 10 µM DTG exposure solutions, we chose to use fish water with 0.1% final DMSO concentration in all described experiments, although the 15 and 20 µM DTG final exposure concentration solutions used for experiments evaluating survival rates and gross morphological effects contained more than 0.1% final DMSO concentration (precisely 0.15% and 0.2% final DMSO concentrations, respectively). We chose the 0.1% DMSO concentration for the negative control and for 1 and 10 µM DTG solutions, since further more impactful experiments described in Section 2.2, Section 2.3, Section 2.4 and Section 2.5 of the Results section on neurodevelopmental and behavioral effects of DTG exposure were performed only with the 1 µM DTG concentration (where the 0.1% DMSO was used for both DTG-exposed and control groups) and because DMSO concentrations up to 1% are described to be safe when used in the zebrafish embryo developmental toxicity assay and up to 0.55% do not impact larval behavior [78,79]. Alive embryos at different stages, according to each described experiment, were dechorionated to maximize the drug uptake (with the exception of embryos used for hatching rate evaluation), transferred to glass Petri dishes and exposed to DTG at the selected concentrations in the 1–20 µM range (1–10–15–20 µM) by the classic static immersion method [80], from 4 hpf or from 6 hpf and up to 16, 24, 40, 48, 72 or 144 hpf, depending on the different type of further analyses (Figure 1). The DTG concentration range was chosen considering the established human C_min_-C_max_ interval for DTG (3–10 μM) [10] and that, in our previous experiments, the exposure of embryos above a 2× concentration of the human C_max_ (i.e., above 20 µM) resulted in such effects on embryo morphology and embryo survival that were too severe to allow further morphological and molecular analyses. Initial experiments (survival and morphology experiments) were conducted with all DTG concentrations; for further experiments, only the lowest 1 µM DTG concentration was chosen. DTG exposure start time (4 or 6 hpf) was chosen on the basis of previous observations [10]; exposure start time at 72 hpf was used only for comparison analysis of locomotor behavior (see below and Figure 1). For experiments with FA, we used the supplementation concentration of 60 ng/mL, as previously described by Cabrera and colleagues [10]. In distinct experiments, FA was supplemented before DTG treatment (2 hpf) or after DTG treatment (10 hpf) to differently evaluate its effect in preventing or rescuing DTG exposure effects, respectively. In further experiments designed to further investigate larvae behavior after DTG exposure, FA was added as above at the 10 hpf stage and then DTG was added at 72 hpf (see experimental exposure and analysis timelines in Figure 1). As negative control, for all experiments, embryos were exposed to 0.1% DMSO in fish water (expected mortality rate < 10%) [81,82].

### 4.4. Evaluation of Mortality and Gross Developmental Morphology

Drug-induced mortality and embryotoxicity were evaluated by the Fish Embryo Toxicity test (FET), essentially as previously described [80,82,83]. Survival rate at 48 hpf was recorded after DTG exposure (1–10–15–20 µM) at 6 hpf. Survival rate at 48 hpf was recorded after DTG exposure (1–10–15–20 µM) at 4 hpf. Further experiments evaluated survival rate at 144 hpf after 1 µM DTG exposure at 4 hpf. For experiments with FA added before 1 µM DTG exposure, i.e., 2 hpf, or after 1 µM DTG exposure, i.e., at 10 hpf, survival rate was also evaluated at 48 and 144 hpf. Dose–response graphs were plotted for all experiments. Morphological observations after DTG exposure at 6 hpf were performed for all tested concentrations at 48 hpf. For morphology analyses after DTG exposure at 4 hpf, we chose only the 1 µM concentration, since phenotypic alterations were already evident at that concentration. In this case, morphology was evaluated at both 24 and 48 hpf. For experiments with FA added before 1 µM DTG exposure, i.e., 2 hpf, or after 1 µM DTG exposure, i.e., at 10 hpf, morphology was evaluated at both 24 and 48 hpf, like above, and further at 144 hpf. In all experiments, morphology was carefully evaluated by visual inspection from head to tail of embryos, anesthetized with 0.4% Tricaine (Sigma-Aldrich). For analyses, microscope direct visualization was employed with a Zeiss Axiozoom V13 microscope (Carl Zeiss AG, Oberkochen, Germany), equipped with PlanNeoFluar Z 1×/0.25 FWD 56 mm lens, and images were analyzed with Zen 3.5 software Blue version (Carl Zeiss AG) (magnification 20×). As developmental indicators, we considered gross morphological changes like alterations in the head regions (particularly main brain areas like forebrain, midbrain, hindbrain, ventricle regions, midbrain–hindbrain boundary length and eye diameter), tail morphology, maintenance of a correct anterior–posterior (AP) axis or its deformation and normal growth or growth delay (body length). Body length, eye diameter and midbrain–hindbrain boundary length measurements for control and 1 µM DTG-exposed embryos were performed on digital images using the ImageJ Fiji software (https://imagej.nih.gov/ij/, accessed on 13 February 2024), as previously described [75] and considering 10 embryos for each experimental condition and for each of three experiments (a total of 30 embryos). The phenotype of the embryos after drug exposure at the blastula stage was then classified as normal, mild or severe, based on the abnormality of the developmental morphological endpoints that are described in Table 1. Embryos were defined with a mild or a severe morphological phenotype in comparison with normal untreated embryos when harboring at least one of the developmental defects described in Table 1. Percentages of malformed larvae with a mild or severe phenotype after drug exposure at the blastula stage were recorded at 24 and 48 hpf for the lowest 1 µM DTG concentration. Body length, eye diameter and midbrain–hindbrain boundary length measurements were further performed on digital images as above for experiments also with FA supplementation (pre- and post-exposure), considering again 10 embryos for each experimental condition and for each of three experiments (a total of 30 embryos). Each experiment for the evaluation of both mortality and gross developmental morphology was repeated at least three times for both the control and the treated groups, with at least 30 embryos for each group and each experiment.

### 4.5. Whole-Mount In Situ Hybridization (WISH)

All experiments were performed with embryos treated with DTG 1 µM, with or without FA supplementation at 10 hpf. Digoxigenin (DIG)-labelled antisense RNA probes for *no tail* (*ntl*), *paired box 2a* (*pax2a*), *neurogenin1* (*ngn1*) and *neuronal differentiation 1* (*neurod1*) genes were prepared by in vitro transcribed linearized cDNA clones with T7 and SP6 polymerase using DIG Labeling Mix (Roche, Basel, Switzerland), essentially as previously described [80]. Whole-mount in situ hybridization (WISH) experiments were performed according to standard methods, as previously described [80]. Briefly, treated embryos, at different developmental stages (hpf) depending on each gene expression evaluation (Figure 1), were fixed overnight in 4% (*v*/*v*) paraformaldehyde (Sigma-Aldrich) at 4 °C, dehydrated through an ascending methanol (Sigma-Aldrich) series and stored at −20 °C. After permeabilization with 10 µg/mL proteinase K (Roche), embryos were hybridized overnight at 68 °C with DIG-labelled antisense RNA probes (400 ng). After repeated washes at high stringent temperature with saline-sodium citrate buffer (SSC) 2×/phosphate-buffered saline (PBS) 1× and SSC 0·2×/PBS 1×, embryos were incubated with anti-DIG antibody (1:10,000) conjugated with alkaline phosphatase (Roche) overnight at 4 °C. Staining was performed with nitro blue tetrazolium (NBT)/5-bromo-4-chloro-3-indolyl-phosphate (BCIP) (Roche) alkaline phosphatase substrates, according to the manufacturer’s instructions. Embryos were mounted in agarose-coated dishes, and WISH images were taken with a Leica MZ16F stereomicroscope equipped with DFC 480 digital camera and LAS Leica Imaging software (Version 5.1.0 Leica Microsystems, Wetzlar, Germany) at 20× magnification. Each WISH experiment was repeated three times for both the control and the treated embryos, with 20 embryos for each group and each experiment.

### 4.6. Image Analysis of Neurogenin 1 (ngn1) Expression with Tg(ngn1:EGFP) Line

Experiments were performed with embryos treated with DTG 1 µM, with or without FA supplementation at 10 hpf, or fish water plus 0.1% DMSO as the control. *ngn1* expression was visualized in live transgenic Tg(*ngn1*:EGFP) embryos at 40 hpf. While *ngn1* expression by WISH was evaluated at 24 hpf since the transcription factor is mainly and transiently expressed as mRNA at this developmental stage, we choose to examine transgenic embryos 16 h late, since (*ngn1* promoter driven-)-EGFP signal is more stable than *ngn1* signal and due to the physiological delay between transcription and translation. Further, in zebrafish, motor neurons start forming at 34 hpf, while complete motor neuron projections are visible at 40 hpf. At 40 hpf, embryos were anesthetized with 0.4% Tricaine and mounted in 1% low melting agarose gel. Fluorescent images were acquired for the control and treated groups. The images were taken in lateral and dorsal position at 20× magnification with Zeiss Axiozoom V13 fluorescence microscope (Carl Zeiss AG), equipped with PlanNeoFluar Z 1×/0.25 FWD 56 mm lens and processed with Zen 3.5 Blue version (Carl Zeiss AG). Spinal cord neurons and spinal motor neuron projections were visualized at 40× magnification. Each experiment was repeated three times for both the control and the treated embryos, with 25 embryos for each group and each experiment. Measurements for the midbrain size (width), cranial ganglia distance (right-to-left cranial ganglia) and anterior hindbrain size (width) were analyzed on dorsal view images using ImageJ Fiji software, in three distinct experiments and in 10 embryos per treatment.

### 4.7. Hatching Rate

For hatching rate determination, non-dechorionated eggs were used and treated as above, always considering the 1 µM DTG concentration at 4 hpf, with or without FA supplementation at 10 hpf, or fish water plus 0.1% DMSO as the control. Hatching rate was recorded at 72 hpf and plotted as the percentage of hatched larvae. Each hatching rate experiment was repeated three times for both the control and the treated embryos, with 25 embryos for each group and each experiment.

### 4.8. Behavior Assessment by the Light–Dark Locomotion and Touch-Evoked Tests

Embryos were exposed at the blastula stage (4 hpf) to 1 μM DTG, with or without 60 ng/mL FA at 10 hpf, or fish water plus 0.1% DMSO as the control, until 144 hpf. In further experiments, embryos were exposed at 72 hpf to 1 μM DTG, previously supplemented with 60 ng/mL FA at 10 hpf or not supplemented, or fish water plus 0.1% DMSO as the control, until 144 hpf. The light–dark locomotion test was performed essentially as previously described [80]. Briefly, for each treatment, 12 alive larvae at 144 hpf were collected in a 96-square well plate with one larva per well in a volume of 200 µL. The 96-square well plate was then put in the observation chamber of the *Danio Vision* Noldus system holder (Noldus, Wageningen, The Netherlands), in an isolated noise-free room. Larvae were allowed to adapt for 30 min before video recording. The system was then set up to track movements (moved distance in 2 min time bins) for 2 h during 6 cycles of alternating light and dark 10 min periods. Data were analyzed using the Noldus *Ethovision* software (Version XT 13.0). Movements were reported as total distance (cm) travelled by the larvae and speed as mm/s, calculated under both light and dark stimuli. Each light–dark locomotion experiment was repeated three times for both the control and the treated embryos, with 12 embryos for each group and each experiment. For the touch-evoked test, embryos were treated as above (DTG 1 μM at 4 hpf with or without 60 ng/mL FA at 10 hpf, or fish water plus 0.1% DMSO as the control) until 72 hpf. At this stage, 20 embryos for each treatment and each of three experiments were subjected to the touch-evoked test. Briefly, embryos were transferred into Petri dishes containing the respective test solutions. A motility wheel consisting of two concentric circles of increasing diameter (20 mm and >20 mm) was placed under the microscope and centered at the bottom of the Petri dish. Each embryo was placed at the center of the Petri dish and the tail was gently touched with a smooth pipette tip. Touch response was observed and categorized as follows: (1) embryos that did not move; (2) embryos that swam < 20 mm; and (3) embryos that crossed the inner circle and swam > 20 mm. Percentages of each category for each treatment were calculated.

### 4.9. Tyrosine Hydroxylase Immunofluorescence Experiments

Experiments were performed with embryos treated with DTG 1 µM, with or without FA supplementation at 10 hpf, or fish water plus 0.1% DMSO as the control. For tyrosine hydroxylase (TH) immunofluorescence, 48 hpf embryos were fixed in in 4% (*v*/*v*) paraformaldehyde for two hours at room temperature, then rinsed twice in PBS for 1 h. Embryos were permeabilized in 10% methanol (Sigma-Aldrich) in PBS for 1 h under gentle agitation. Non-specific binding was blocked overnight in 10% Normal Sheep Serum (NSS) (Sigma-Aldrich), 1% bovine serum albumin (BSA) (Sigma-Aldrich) in PBS1×/0.3%Triton X-100 (Sigma-Aldrich). Embryos were then incubated for 72 h with rabbit anti-TH (1:200, ab152, Merck Millipore, Burlington, MA, USA) primary antibody in the blocking solution at 4 °C. After three washes in PBS 1× for 1 h, samples were incubated with the anti-rabbit Cy3 (1:100, 111-165-144, Jackson Immunoresearch, Cambridge, UK) secondary antibody diluted in 10% NSS, 1% BSA in PBS 1× for 40 h at 4 °C and then rinsed several times in PBS 1×. TH-stained embryos were visualized using Zeiss Axio Observer microscope (Carl Zeiss AG) (magnification 20×) and analyzed using Zen 3.5 (blue Version) software (Carl Zeiss AG). Each experiment was repeated three times for both the control and the treated embryos, with 20 embryos for each group and each experiment. A defined area (white box) was analyzed to count the number of TH^+^ neurons, using Zen 3.5 (blue Version) software (Carl Zeiss AG). Count was performed on all embryos.

### 4.10. Statistical Analysis

All graphs were plotted and analyzed using GraphPad Prism 8 (GraphPad Software). Significance was analyzed by the 1-way ANOVA test with post hoc Tukey’s multiple comparison test. In all cases, *p*-values less than 0.05 were considered as statistically significant (* *p* < 0.05; ** *p* < 0.005; *** *p* < 0.001; **** *p* < 0.0001).

## 5. Conclusions

In conclusion, given our findings, we further emphasize the need for monitoring FA levels in women on DTG therapy, considering FA supplementation especially in women of child-bearing age. Moreover, we raise the need for assessing the potential long-term impacts of DTG in children exposed during pregnancy, focusing on both cognitive and motorial activities, particularly when born to mothers without FA dietary supplementation. Studying DTG neurotoxicity in in vitro and in vivo animal models may help to understand the mechanisms involved and comprehend long-term effects observed in adult patients.

## Figures and Tables

**Table 1:** Criteria for the grading of embryos into normal, mild and severe phenotype.

Morphological Endpoints	Normal	Mild	Severe
Head	Flexed	Straight	Extended
Tail	Properly detached and straight	Slightly shorter length and curved	Shorter length and curved
Anterior posterior (A-P) axis	Normal head and tail position	AP axis mildly disrupted	AP axis not well formed
Somites	V-shaped	U-shaped	Straight

## Data Availability

The data that support the findings of this study are available from the corresponding author upon reasonable request.

## Supplementary Materials

The following supporting information can be downloaded at: https://www.mdpi.com/article/10.3390/ijms25094640/s1.

## References

[1] Scarsi K.K., Havens J.P., Podany A.T., Avedissian S.N., Fletcher C.V. (2020). HIV-1 Integrase Inhibitors: A Comparative Review of Efficacy and Safety. Drugs.

[2] Teira R., Diaz-Cuervo H., Aragão F., Marguet S., de la Fuente B., Muñoz M.J., Abdulghani N., Ribera E., Domingo P., Deig E. (2021). Real world effectiveness of standard of care triple therapy versus two-drug combinations for treatment of people living with HIV. PLoS ONE.

[3] Ciccullo A., Baldin G., Borghi V., Lagi F., Latini A., D’ettorre G., Oreni L., Fusco P., Capetti A., Fabbiani M. (2022). Real-Life Impact of Drug Toxicity on Dolutegravir Tolerability: Clinical Practice Data from a Multicenter Italian Cohort. Viruses.

[4] Zash R., Makhema J., Shapiro R.L. (2018). Neural-Tube Defects with Dolutegravir Treatment from the Time of Conception. N. Engl. J. Med..

[5] Zash R., Holmes L., Diseko M., Jacobson D.L., Brummel S., Mayondi G., Isaacson A., Davey S., Mabuta J., Mmalane M. (2019). Neural-Tube Defects and Antiretroviral Treatment Regimens in Botswana. N. Engl. J. Med..

[6] Pereira G.F.M., Kim A., Jalil E.M., Fonseca F.F., E Shepherd B., Veloso V.G., Rick F., Ribeiro R., Pimenta M.C., Beber A. (2021). Dolutegravir and pregnancy outcomes in women on antiretroviral therapy in Brazil: A retrospective national cohort study. Lancet HIV.

[7] Kourtis A.P., Zhu W., Lampe M.A., Huang Y.A., Hoover K.W. (2023). Dolutegravir and pregnancy outcomes including neural tube defects in the USA during 2008–2020: A national cohort study. Lancet HIV.

[8] Crowell C.S., Williams P.L., Yildirim C., Van Dyke R.B., Smith R., Chadwick E.G., Seage G.R., Diperna A., Hazra R. (2020). Safety of in-utero antiretroviral exposure: Neurologic outcomes in children who are HIV-exposed but uninfected. AIDS.

[9] (2021). NEJM Journal Watch. https://www.jwatch.org/na53453/2021/04/23/antiretroviral-therapy-pregnant-women-with-hiv-safe-and.

[10] Cabrera R.M., Souder J.P., Steele J.W., Yeo L., Tukeman G., Gorelick D.A., Finnell R.H. (2019). The antagonism of folate receptor by dolutegravir: Developmental toxicity reduction by supplemental folic acid. AIDS.

[11] Gilmore J.C., Hoque T., Dai W., Mohan H., Dunk C., Serghides L., Bendayan R. (2021). Interaction between dolutegravir and folate transporters and receptor in human and rodent placenta. EBioMedicine.

[12] Mohan H., Nguyen J., MacKenzie B., Yee A., Laurette E.Y., Sanghvi T., Tejada O., Dontsova V., Leung K.-Y., Goddard C. (2023). Folate deficiency increases the incidence of dolutegravir-associated foetal defects in a mouse pregnancy model. eBioMedicine.

[13] Small C.D., Crawford B.D. (2016). Matrix metalloproteinases in neural development: A phylogenetically diverse perspective. Neural Regen. Res..

[14] Bade A.N., McMillan J.M., Liu Y., Edagwa B.J., Gendelman H.E. (2021). Dolutegravir Inhibition of Matrix Metalloproteinases Affects Mouse Neurodevelopment. Mol. Neurobiol..

[15] Basnet R.M., Zizioli D., Taweedet S., Finazzi D., Memo M. (2019). Zebrafish Larvae as a Behavioral Model in Neuropharmacology. Biomedicines.

[16] Schmidt R., Strähle U., Scholpp S. (2013). Neurogenesis in zebrafish-from embryo to adult. Neural Dev..

[17] Schnoll J.G., Temsamrit B., Zhang D., Song H., Ming G.L., Christian K.M. (2021). Evaluating Neurodevelopmental Consequences of Perinatal Exposure to Antiretroviral Drugs: Current Challenges and New Approaches. J. Neuroimmune Pharmacol..

[18] Foster E.G., Gendelman H.E., Bade A.N. (2022). HIV-1 Integrase Strand Transfer Inhibitors and Neurodevelopment. Pharmaceuticals.

[19] Lambert A.M., Bonkowsky J.L., Masino M.A. (2012). The conserved dopaminergic diencephalospinal tract mediates vertebrate locomotor development in zebrafish larvae. J. Neurosci..

[20] Reimer M.M., Norris A., Ohnmacht J., Patani R., Zhong Z., Dias T.B., Kuscha V., Scott A.L., Chen Y.-C., Rozov S. (2013). Dopamine from the brain promotes spinal motor neuron generation during development and adult regeneration. Dev. Cell.

[21] Iannetta A., Caioni G., Di Vito V., Benedetti E., Perugini M., Merola C. (2022). Developmental toxicity induced by triclosan exposure in zebrafish embryos. Birth Defects Res..

[22] Selderslaghs I.W., Hooyberghs J., Blust R., Witters H.E. (2013). Assessment of the developmental neurotoxicity of compounds by measuring locomotor activity in zebrafish embryos and larvae. Neurotoxicol. Teratol..

[23] de Oliveira A.A.S., Brigante T.A.V., Oliveira D.P. (2021). Tail Coiling Assay in Zebrafish (Danio rerio) Embryos: Stage of Development, Promising Positive Control Candidates, and Selection of an Appropriate Organic Solvent for Screening of Developmental Neurotoxicity (DNT). Water.

[24] Kimmel C.B., Patterson J., Kimmel R.O. (1974). The development and behavioral characteristics of the startle response in the zebra fish. Dev. Psychobiol..

[25] Money D., Lee T., O’Brien C., Brophy J., Bitnun A., Kakkar F., Boucoiran I., Alimenti A., Vaudry W., Singer J. (2019). Canadian Perinatal HIV Surveillance Program. Congenital anomalies following antenatal exposure to dolutegravir: A Canadian surveillance study. BJOG Int. J. Obstet. Gynaecol..

[26] Mohan H., Lenis M.G., Laurette E.Y., Tejada O., Sanghvi T., Leung K.-Y., Cahill L.S., Sled J.G., Delgado-Olguín P., Greene N.D. (2021). Dolutegravir in pregnant mice is associated with increased rates of fetal defects at therapeutic but not at supratherapeutic levels. EBioMedicine.

[27] Tukeman G.L., Wei H., Finnell R.H., Cabrera R.M. (2024). Dolutegravir induced neural tube defects in mice are folate responsive. AIDS.

[28] Smith C., Fought A.J., Sung J.F., McKinney J.R., Metz T.D., Fetters K.B., Lazarus S., Capraro S., Barr E., Glenny C. (2023). Congenital malformations and preeclampsia associated with integrase inhibitor use in pregnancy: A single-center analysis. PLoS ONE.

[29] Kala S., Watson B., Zhang J.G., Papp E., Lenis M.G., Dennehy M., Cameron D.W., Harrigan P.R., Serghides L. (2018). Improving the clinical relevance of a mouse pregnancy model of antiretroviral toxicity; a pharmacokinetic dosing-optimization study of current HIV antiretroviral regimens. Antivir. Res..

[30] Cassar S., Beekhuijzen M., Beyer B., Chapin R., Dorau M., Hoberman A., Krupp E., Leconte I., Stedman D., Stethem C. (2019). A multi-institutional study benchmarking the zebrafish developmental assay for prediction of embryotoxic plasma concentrations from rat embryo-fetal development studies. Reprod. Toxicol..

[31] Gibbs H.C., Chang-Gonzalez A., Hwang W., Yeh A.T., Lekven A.C. (2017). Midbrain-Hindbrain Boundary Morphogenesis: At the Intersection of Wnt and Fgf Signaling. Front. Neuroanat..

[32] Scholpp S., Brand M. (2003). Integrity of the midbrain region is required to maintain the diencephalic-mesencephalic boundary in zebrafish no isthmus/pax2.1 mutants. Dev. Dyn..

[33] Montenegro-Burke J.R., Woldstad C.J., Fang M., Bade A.N., McMillan J., Edagwa B., Boska M.D., Gendelman H.E., Siuzdak G. (2019). Nanoformulated Antiretroviral Therapy Attenuates Brain Metabolic Oxidative Stress. Mol. Neurobiol..

[34] Foster E.G., Sillman B., Liu Y., Summerlin M., Kumar V., Sajja B.R., Cassidy A.R., Edagwa B., Gendelman H.E., Bade A.N. (2023). Long-acting dolutegravir formulations prevent neurodevelopmental impairments in a mouse model. Front. Pharmacol..

[35] Smith M.R., Mohan H., Ajaykumar A., Hsieh A.Y.Y., Martineau L., Patel R., Gadawska I., Sherwood C., Serghides L., Piret J.M. (2022). Second-Generation Human Immunodeficiency Virus Integrase Inhibitors Induce Differentiation Dysregulation and Exert Toxic Effects in Human Embryonic Stem Cell and Mouse Models. J. Infect. Dis..

[36] Prado L.I.A., Junger A.L., Caixeta L.F., Noll M., Oliveira C., Silveira É.A. (2023). The Effects of Methylfolate on Cognitive Decline and Dementia: A Protocol for Systematic Review and Meta-Analysis. J. Clin. Med..

[37] Kishida R., Yamagishi K., Ikeda A., Hayama-Terada M., Shimizu Y., Muraki I., Umesawa M., Imano H., Sankai T., Okada T. (2024). Serum folate and risk of disabling dementia: A community-based nested case-control study. Nutr. Neurosci..

[38] Kimmel C.B. (1989). Genetics and early development of zebrafish. Trends Genet..

[39] Pinheiro D., Heisenberg C.P. (2020). Zebrafish gastrulation: Putting fate in motion. Curr. Top. Dev. Biol..

[40] Rai S., Leydier L., Sharma S., Katwala J., Sahu A. (2023). A quest for genetic causes underlying signaling pathways associated with neural tube defects. Front. Pediatr..

[41] Wang X., Yu J., Wang J. (2023). Neural Tube Defects and Folate Deficiency: Is DNA Repair Defective?. Int. J. Mol. Sci..

[42] Amacher S.L., Draper B.W., Summers B.R., Kimmel C.B. (2002). The zebrafish T-box genes no tail and spadetail are required for development of trunk and tail mesoderm and medial floor plate. Development.

[43] Marlow F., Gonzalez E.M., Yin C., Rojo C., Solnica-Krezel L. (2004). No tail co-operates with non-canonical Wnt signaling to regulate posterior body morphogenesis in zebrafish. Development.

[44] Morley R.H., Lachani K., Keefe D., Gilchrist M.J., Flicek P., Smith J.C., Wardle F.C. (2009). A gene regulatory network directed by zebrafish No tail accounts for its roles in mesoderm formation. Proc. Natl. Acad. Sci. USA.

[45] Harvey S.A., Tümpel S., Dubrulle J., Schier A.F., Smith J.C. (2010). No tail integrates two modes of mesoderm induction. Development.

[46] Schulte-Merker S., van Eeden F.J., Halpern M.E., Kimmel C.B., Nüsslein-Volhard C. (1994). No tail (ntl) is the zebrafish homologue of the mouse T (Brachyury) gene. Development.

[47] Yamakoshi K., Shimoda N. (2003). De novo DNA methylation at the CpG island of the zebrafish no tail gene. Genesis.

[48] Chang S., Lu X., Wang S., Wang Z., Huo J., Huang J., Shangguan S., Li S., Zou J., Bao Y. (2019). The effect of folic acid deficiency on FGF pathway via Brachyury regulation in neural tube defects. FASEB J..

[49] Lee M.S., Bonner J.R., Bernard D.J., Sanchez E.L., Sause E.T., Prentice R.R., Burgess S.M., Brody L.C. (2012). Disruption of the folate pathway in zebrafish causes developmental defects. BMC Dev. Biol..

[50] Macdonald R., Scholes J., Strähle U., Brennan C., Holder N., Brand M., Wilson S.W. (1997). The Pax protein Noi is required for commissural axon pathway formation in the rostral forebrain. Development.

[51] Blader P., Fischer N., Gradwohl G., Guillemot F., Strähle U. (1997). The activity of neurogenin1 is controlled by local cues in the zebrafish embryo. Development.

[52] Blader P., Plessy C., Strähle U. (2003). Multiple regulatory elements with spatially and temporally distinct activities control neurogenin1 expression in primary neurons of the zebrafish embryo. Mech. Dev..

[53] Jeong J.-Y., Einhorn Z., Mercurio S., Lee S., Lau B., Mione M., Wilson S.W., Guo S. (2006). Neurogenin1 is a determinant of zebrafish basal forebrain dopaminergic neurons and is regulated by the conserved zinc finger protein Tof/Fezl. Proc. Natl. Acad. Sci. USA.

[54] Korzh V., Sleptsova I., Liao J., He J., Gong Z. (1998). Expression of zebrafish bHLH genes ngn1 and nrd defines distinct stages of neural differentiation. Dev. Dyn..

[55] Mueller T., Wullimann M.F. (2002). Expression domains of neuroD (nrd) in the early postembryonic zebrafish brain. Brain Res. Bull..

[56] Tay T.L., Ronneberger O., Ryu S., Nitschke R., Driever W. (2011). Comprehensive catecholaminergic projectome analysis reveals single-neuron integration of zebrafish ascending and descending dopaminergic systems. Nat. Commun..

[57] Kastenhuber E., Kratochwil C.F., Ryu S., Schweitzer J., Driever W. (2010). Genetic dissection of dopaminergic and noradrenergic contributions to catecholaminergic tracts in early larval zebrafish. J. Comp. Neurol..

[58] Shaikh A., Roy H. (2023). Folate deprivation induced neuroinflammation impairs cognition. Neurosci. Lett..

[59] Meiser J., Weindl D., Hiller K. (2013). Complexity of dopamine metabolism. Cell Commun. Signal..

[60] Murray L.K., Jadavji N.M. (2019). The role of one-carbon metabolism and homocysteine in Parkinson’s disease onset, pathology and mechanisms. Nutr. Res. Rev..

[61] Duan W., Ladenheim B., Cutler R.G., Kruman I.I., Cadet J.L., Mattson M.P. (2002). Dietary folate deficiency and elevated homocysteine levels endanger dopaminergic neurons in models of Parkinson’s disease. J. Neurochem..

[62] Lee E.S., Chen H., Soliman K.F., Charlton C.G. (2005). Effects of homocysteine on the dopaminergic system and behavior in rodents. Neurotoxicology.

[63] Thöny B., Auerbach G., Blau N. (2000). Tetrahydrobiopterin biosynthesis, regeneration and functions. Biochem. J..

[64] Moens A.L., Kass D.A. (2006). Tetrahydrobiopterin and cardiovascular disease. Arterioscler. Thromb. Vasc. Biol..

[65] Cornell R.A., Eisen J.S. (2002). Delta/Notch signaling promotes formation of zebrafish neural crest by repressing Neurogenin 1 function. Development.

[66] Katz H.R., Menelaou E., Hale M.E. (2021). Morphological and physiological properties of Rohon-Beard neurons along the zebrafish spinal cord. J. Comp. Neurol..

[67] Shane B., Stokstad E.L. (1985). Vitamin B12-folate interrelationships. Annu. Rev. Nutr..

[68] Morris D.R., Levenson C.W. (2013). Zinc regulation of transcriptional activity during retinoic acid-induced neuronal differentiation. J. Nutr. Biochem..

[69] Sutherland M.J., Wang S., Quinn M.E., Haaning A., Ware S.M. (2013). Zic3 is required in the migrating primitive streak for node morphogenesis and left-right patterning. Hum. Mol. Genet..

[70] Winata C.L., Kondrychyn I., Kumar V., Srinivasan K.G., Orlov Y., Ravishankar A., Prabhakar S., Stanton L.W., Korzh V., Mathavan S. (2013). Genome wide analysis reveals Zic3 interaction with distal regulatory elements of stage specific developmental genes in zebrafish. PLoS Genet..

[71] Jiang Y.G., Wang Y.H., Zhang H., Wang Z.Y., Liu Y.Q. (2022). Effects of early-life zinc deficiency on learning and memory in offspring and the changes in DNA methylation patterns. Nutr. Neurosci..

[72] Kirkwood-Johnson L., Katayama N., Marikawa Y. (2021). Dolutegravir Impairs Stem Cell-Based 3D Morphogenesis Models in a Manner Dependent on Dose and Timing of Exposure: An Implication for Its Developmental Toxicity. Toxicol. Sci..

[73] Ramesh T., Nagula S.V., Tardieu G.G., Saker E., Shoja M., Loukas M., Oskouian R.J., Tubbs R.S. (2017). Update on the notochord including its embryology, molecular development, and patology: A primer for the clinician. Cureus.

[74] Corallo D., Trapani V., Bonaldo P. (2015). The notochord: Structure and functions. Cell. Mol. Life Sci..

[75] Zizioli D., Ferretti S., Mignani L., Castelli F., Tiecco G., Zanella I., Quiros-Roldan E. (2023). Developmental safety of nirmatrelvir in zebrafish (Danio rerio) embryos. Birth Defects Res..

[76] Kimmel C.B., Ballard W.W., Kimmel S.R., Ullmann B., Schilling T.F. (1995). Stages of embryonic development of the zebrafish. Dev. Dyn..

[77] DrugBank Online. https://go.drugbank.com/salts/DBSALT000943.

[78] Christou M., Kavaliauskis A., Ropstad E., Fraser T.W.K. (2020). DMSO effects larval zebrafish (Danio rerio) behavior, with additive and interaction effects when combined with positive controls. Sci. Total Environ..

[79] Hoyberghs J., Bars C., Ayuso M., Van Ginneken C., Foubert K., Van Cruchten S. (2021). DMSO Concentrations up to 1% are Safe to be Used in the Zebrafish Embryo Developmental Toxicity Assay. Front. Toxicol..

[80] Zizioli D., Zanella I., Mignani L., Degli Antoni M., Castelli F., Quiros-Roldan E. (2023). Cabotegravir Exposure of Zebrafish (Danio rerio) Embryos Impacts on Neurodevelopment and Behavior. Int. J. Mol. Sci..

[81] von Hellfeld R., Brotzmann K., Baumann L., Strecker R., Braunbeck T. (2020). Adverse effects in the fish embryo acute toxicity (FET) test: A catalogue of unspecific morphological changes versus more specific effects in zebrafish (Danio rerio) embryos. Environ. Sci. Eur..

[82] (2013). OECDiLibrary. https://www.oecd-ilibrary.org/environment/test-no-236-fish-embryo-acute-toxicity-fet-test_9789264203709-en.

[83] Zizioli D., Ferretti S., Tiecco G., Mignani L., Monti E., Castelli F., Quiros-Roldan E., Zanella I. (2023). Comparison of Efavirenz and Doravirine Developmental Toxicity in an Embryo Animal Model. Int. J. Mol. Sci..

