# Supplementary material for: Dolutegravir and Folic Acid Interaction during Neural System Development in Zebrafish Embryos
Source: Int J Mol Sci. 2024 Apr 24;25(9):4640. doi: 10.3390/ijms25094640 (PMC11083492; doi:10.3390/ijms25094640)
Supplement: Supplementary file 1 [file ijms-25-04640-s001.zip › ijms-2903280-supplementary.pdf]

Supplementary Material

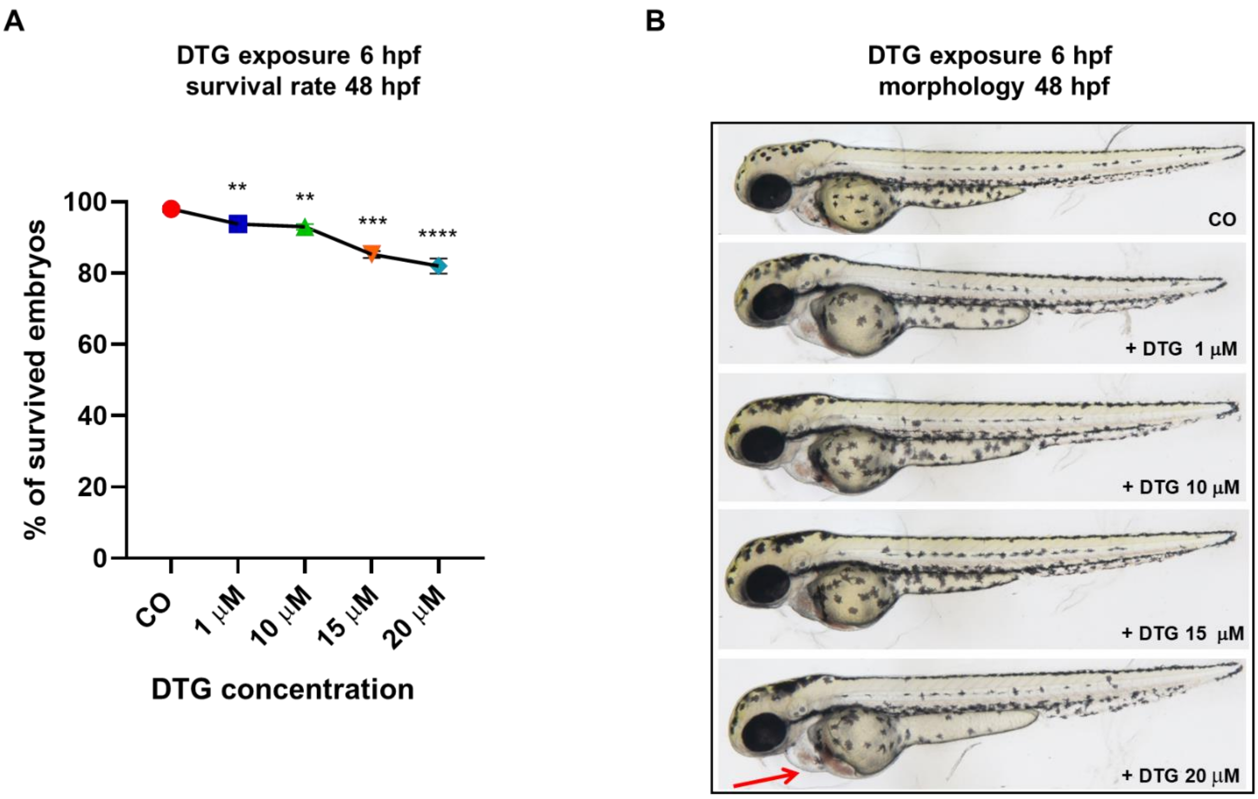

Figure S1. Survival rate and morphology assessment of zebrafish embryos at 48 hours post fertilization (hpf) after exposure to dolutegravir (DTG) at 6 hours post fertilization (hpf) (early gastrulation stage). Dechorionated zebrafish embryos were exposed at early gastrulation stage (6 hpf) to drug solvent only (fish water plus 0.1% dimethyl sulfoxide, DMSO) (CO), or DTG (1-10-15-20 μM), dissolved in fish water. **(A)** Survival rate at 48 hpf of embryos after drug exposure. X-axis shows drug doses used for exposure of embryos; Y-axis shows the corresponding survival percentages. Results are expressed as mean ± SD of three independent experiments, with 30 embryos for each experiment and each treatment. Significance was analyzed by the 1-way ANOVA test with post-hoc Tukey's multiple comparison test (** $p < 0.005$; *** $p < 0.001$, **** $p < 0.0001$). **(B)** Representative pictures at 48 hpf of the gross morphological effects of DTG exposure on zebrafish embryos. All treatments, including controls (CO), were conducted three times, with 30 embryos for each experiment and treatment. Embryos in the figures are representative of all experiments. All pictures are lateral views with dorsal to the top and anterior to the left (magnification 20X). The red arrow indicates the presence of pericardial edema.

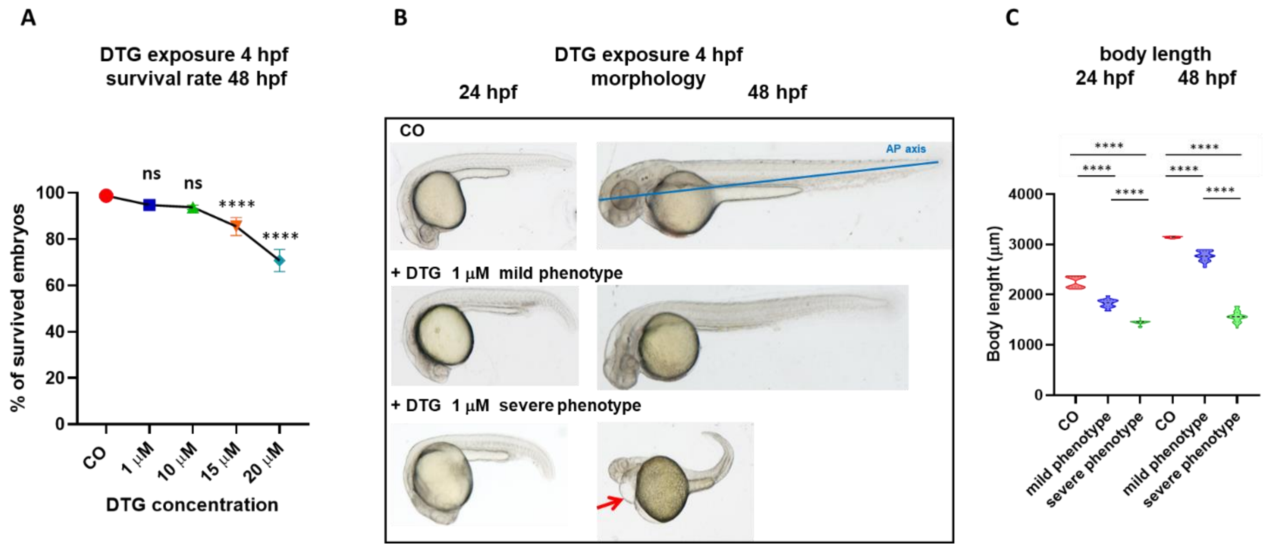

Figure S2. Survival rate at 48 hours post fertilization (hpf), morphology assessment at 24 and 48 hours hpf and body length measures at 24 and 48 hpf of zebrafish embryos after exposure to dolutegravir (DTG) at 4 hpf (blastula stage). Dechorionated zebrafish embryos were exposed at 4 hpf (during the blastula stage) to drug solvent only (fish water plus 0.1% dimethyl sulfoxide, DMSO) (CO), or DTG, dissolved in fish water containing 0.1% DMSO. **(A)** Survival rate at 48 hpf of embryos after drug exposure (DTG, 1-10-15-20 µM). X-axis shows drug doses used for exposure of embryos; Y-axis shows the corresponding survival rate (percentages of survived emryos). Results are expressed as mean ± SD of three independent experiments, with 30 embryos for each experiment and each treatment. Significance was analyzed by the 1-way ANOVA test with post-hoc Tukey's multiple comparison test (ns, statistically not significant; **** $p< 0.0001$). **(B)** Representative pictures at 24 and 48 hpf of the gross morphological effects after 1 µM DTG exposure of zebrafish embryos. Embryos were distinguished in two groups (embryos with mild or severe phenotype), based on the presence of at least one of the morphological defects described in Table 1. All treatments, including controls (CO), were conducted three times, with 30 embryos for each experiment and treatment. Embryos in the figures are representative of all experiments. All pictures are lateral views with dorsal to the top and anterior to the left (magnification 20X). The red arrow indicates the presence of pericardial edema. The blue line indicates the anterior-posterior (AP) axis. **(C)** Violin plot of body length measurements at 24 and 48 hpf for control (CO) and 1 µM DTG-exposed embryos, distinguished as with mild or severe phenotype (X-axis). Y-axis shows body length (µM). Measurements were performed on digital images using the Image J Fiji software [https://imagej.nih.gov/ij/], considering the AP axis, on 10 embryos for each experiment and each treatment (a total of 30 embryos for each experimental condition). Inside each violin the central dashed line marks the median (Q2), the superior and inferior dotted lines mark the third (Q3) and first (Q1) quartiles. Statistical significance amongst and between groups was calculated by the 1-way ANOVA test with post-hoc Tukey's multiple comparison test (**** $p< 0.0001$).

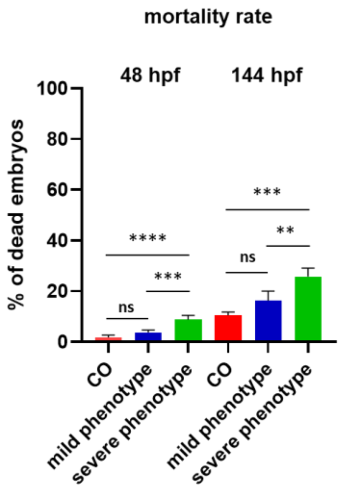

Figure S3. Mortality rate at 48 and 144 hours post fertilization (hpf) of zebrafish embryos after exposure to dolutegravir (DTG) 1 μM at 4 hpf (blastula stage). Dechorionated zebrafish embryos were exposed at 4 hpf (during the blastula stage) to drug solvent only (fish water plus 0.1% dimethyl sulfoxide, DMSO) (CO), or 1 μM DTG, dissolved in fish water containing 0.1% DMSO. Mortality rate was measured at 48 and 144 hpf for control embryos (CO) and DTG treated embryos, distinguished as with mild or severe phenotype (X-axis). Y-axis shows the corresponding percentages of dead embryos. Results are expressed as mean ± SD of three independent experiments, with 30 embryos for each experiment and each treatment. Significance was analyzed by the 1-way ANOVA test with post-hoc Tukey's multiple comparison test (ns, statistically not significant; ** $p< 0.005$; *** $p<0,001$; **** $p< 0.0001$).
